# The benefits of Shuai Shou Gong (SSG) demonstrated in a Randomised Control Trial (RCT) study of older adults in two communities in Thailand

**DOI:** 10.1371/journal.pone.0282405

**Published:** 2023-05-25

**Authors:** Zhen Xiao, Marcos Cruz, Emi Hojo, Wichai Eungpinichpong, Xingze Wang, Li Xiao, Uraiwan Chatchawan, Ying Hu, Neil Roberts

**Affiliations:** 1 Research Center in Back, Neck, Other Joint Pain and Human Performance (BNOJPH), School of Physical Therapy, Faculty of Associated Medical Sciences (AMS), Khon Kaen University (KKU), Khon Kaen, Thailand; 2 Department of Mathematics, Statistics and Computer Science, University of Cantabria, Santander, Spain; 3 Centre for Reproductive Health (CRH), School of Clinical Sciences, University of Edinburgh, Edinburgh, United Kingdom; 4 School of Physical Education, Huzhou University, Huzhou, China; 5 Cardiovascular Medicine, Municipal Hospital, Ganzhou, China; 6 Faculty of Pharmacy, Guangxi University of Chinese Medicine, Nanning, China; PhD, PLOS, UNITED KINGDOM

## Abstract

**Introduction:**

Shuai Shou Gong (SSG) is a type of Arm Swing Exercise (ASE) developed and practiced especially by older people in China for over one thousand years to maintain physical health and well-being. Until now the potential benefits of SSG have not been investigated in a Randomised Control Trial (RCT).

**Materials and methods:**

Fifty six older women were recruited from each of two urban communities in Khon Kaen, Thailand. One community was randomly assigned as the Exercise Group (mean age 68.3 years, standard deviation 5.6 years) and the other as the Control Group (69.4 years, 4.4 years). The Exercise Group performed SSG for 40 minutes, three days per week for two months, whereas the Control Group maintained their usual daily life. Measurements of Posture (C7 to Wall Distance (C7WD), Standing Height (SH), Flexibility (Back Scratch of Left and Right arms (BSL and BSR) and Chair Sit and Reach of Left and Right legs (CSRL and CSRR), Gait (Timed Up and Go (TUG)), and Cognition (Barthel Activities of Daily Living Index (BADL) and Rosenberg Self Esteem Scale (RSES) questionnaires) were recorded for each group prior to, on day 1, week 4, and week 8 of the SSG training.

**Results:**

The 8 week SSG training course produced a significant interaction between group and time for the combined set of all outcome measures (C7WD, SH, BSL, BSR, CSRL, CSRR, TUG, BADL, and BSES) (Modified ANOVA-Type Statistic (MATS) p-value < 0.001) and for the four categories of Posture, Flexibility, Gait, and Cognition (all Wald-Type Statistic (WTS) p-values < 0.05) and in all cases the changes in the Exercise Group were in the direction predicted to be beneficial. No significant interaction effect between time and group was found after either one session or four weeks of SSG training for any of the categories (all WTS p > 0.05) with significant effects only arising after eight weeks (all WTS p < 0.05). Thus although alterations were shown to be increasingly beneficial over time the minimum period required to produce a statistically significant benefit from performing SSG training was 8 weeks. For the Control Group no significant changes were identified for Posture, Flexibility and Cognition however a significant deterioration was observed in TUG (WTS p = 0.003).

**Conclusions:**

SSG is a holistic, gentle, rhythmic, whole body sequence of movements that may be readily learned and enjoyed in a group setting and has been confirmed in an RCT study of older adult females to produce significant benefits in Posture, Flexibility, Gait and Cognition.

## Introduction

Older adults often develop so-called flexed posture, also referred to as slumped posture. The principal features are forward head position, thoracic kyphosis, lumbar lordosis and hip flexion [1], together with rigidity and alterations in gait, including slower walking speed, and shorter stride and step length [2]. These changes can seriously affect daily functioning, health, and quality of life [3, 4], producing such symptoms as increased neck pain, headaches, migrane, tempomandibular joint dysfunction, teeth clenching, decreased neck mobility, pinched nerves, numbness, tingling in arms and hands, muscle spasm and myofascial pain syndrome. Of special interest in the present study is whether forward head posture can be reversed by exercise. In particular, the potential benefits of Shuai Shou Gong (SSG), which is a type of Arm Swing Exercise (ASE), were investigated in a Randomised Control Trial (RCT).

SSG was first mentioned in the book of Dharma Yi Jin Jing more than a thousand years ago and is a branch of traditional Chinese Qi Gong which aims to support the maintenance of physical well-being [5]. In Traditional Chinese Medicine (TCM), SSG is considered to facilitate the circulation of Qi in the body and so nourish the internal organs, promote recovery from illnesses and provide positive benefits to general health. The low-intensity physical SSG exercise [6, 7] consists of a repeated set of easy to learn, relatively small amplitude, slow, rhythmic and coordinated movements of the arms and body [8]. The core muscle contractions produced by rhythmic swinging of the arms and bending of the knees during the performance of SSG can be expected to increase muscle strength and joint range of motion, with likely beneficial effects on posture, flexibility and gait.

Qi Gong exercises such as SSG are additionally considered to be a type of meditation providing benefits for psychological health and well-being [9]. In particular, as will be described in the Methods section below, SSG also has an important cognitive component stemming from the fact the first four arms swings are performed with the participant standing upright but on the last occasion a double arm swing is performed, in synchrony with the bending and then straightening of the legs (see Fig 2).

SSG exercise is gentle and may be readily taken up and practiced by interested groups of older adults within a community. For this reason SSG has frequently been used by researchers interested in studying the benefits of exercise for promoting health in older populations. For example, several studies have been performed to investigate the effect of SSG exercise on the physiology of older adults in community settings [10–14] and also patients with obesity [15], diabetes [16–19] and heart disease [20]. The particular effect of SSG on cognitive function in older subjects has also been investigated [21–23], although it would appear that the protocols used did not always fully and accurately incorporate all the customary elements.

Taken together the combined physical, cognitive and social aspects of SSG make it a particularly potent form of exercise. The primary objective of the present study was to investigate in a randomised controlled trial (RCT) whether a community based two month training program of SSG, performed exactly according to the long-established prescription of TCM, would produce predicted improvements in posture, flexibility, gait and cognition in a group of older women compared to a control group.

## Materials and methods

### Experimental design and setting

The experimental design for the study is a prospective, cluster-based Randomized Controlled Trial (RCT). There are a total of 95 communities in Khon Kaen province, Thailand. The lead investigator ZX opened up discussions with the leaders of ten different communities, to whom they had been introduced by a senior staff member at Khon Kaen University (KKU), regarding whether the community they represented would be interested to participate in the study. The ten communities were relatively close to KKU which would make administration of the SSG training programme more straightforward and were similar in terms of socioeconomic and other demographic factors. The decision was made that as soon as two communities confirmed that they would like to participate the study would proceed. Thus the names of these two communities were each placed inside a sealed envelope and by random selection by ZX the first community to be drawn was assigned to be the Exercise Group and the second community to be the Control Group.

Since, as already mentioned, of special interest in the present study is whether SSG training can have a beneficial effect on forward head posture which develops in many older adults, sample size for the Exercise and Control Groups was chosen based on a previous study of the effect of an intervention which aimed to improve posture. In particular, data obtained by Widberg et al. (2009) [24] in a study to investigate the effect of self and manual mobilization for improving posture in 32 men with ankylosing spondylitis were analysed. Improvement was determined according to whether mobilization produced a significant reduction in the value of C7WD (i.e. the distance from the spinous process of the seventh cervical vertebra to a wall immediately behind the participant). For the Training Group, the pre-training average value of C7WD was 5.2 cm (standard deviation (SD) 1.8 cm) and was reduced to 3.9 cm after the training program (SD 1.4 cm), whereas there was no change in the corresponding values for the Control Group. Based on these findings, if the present study of SSG was to have a power of 90% to detect a similar change with alpha level = 0.01 then there would need to be at least 21 participants in each Group. Thus, to allow for a potential drop-out rate of 25% it was decided to recruit 28 participants to each of the Exercise Group and the Control Group.

**Fig 1:**
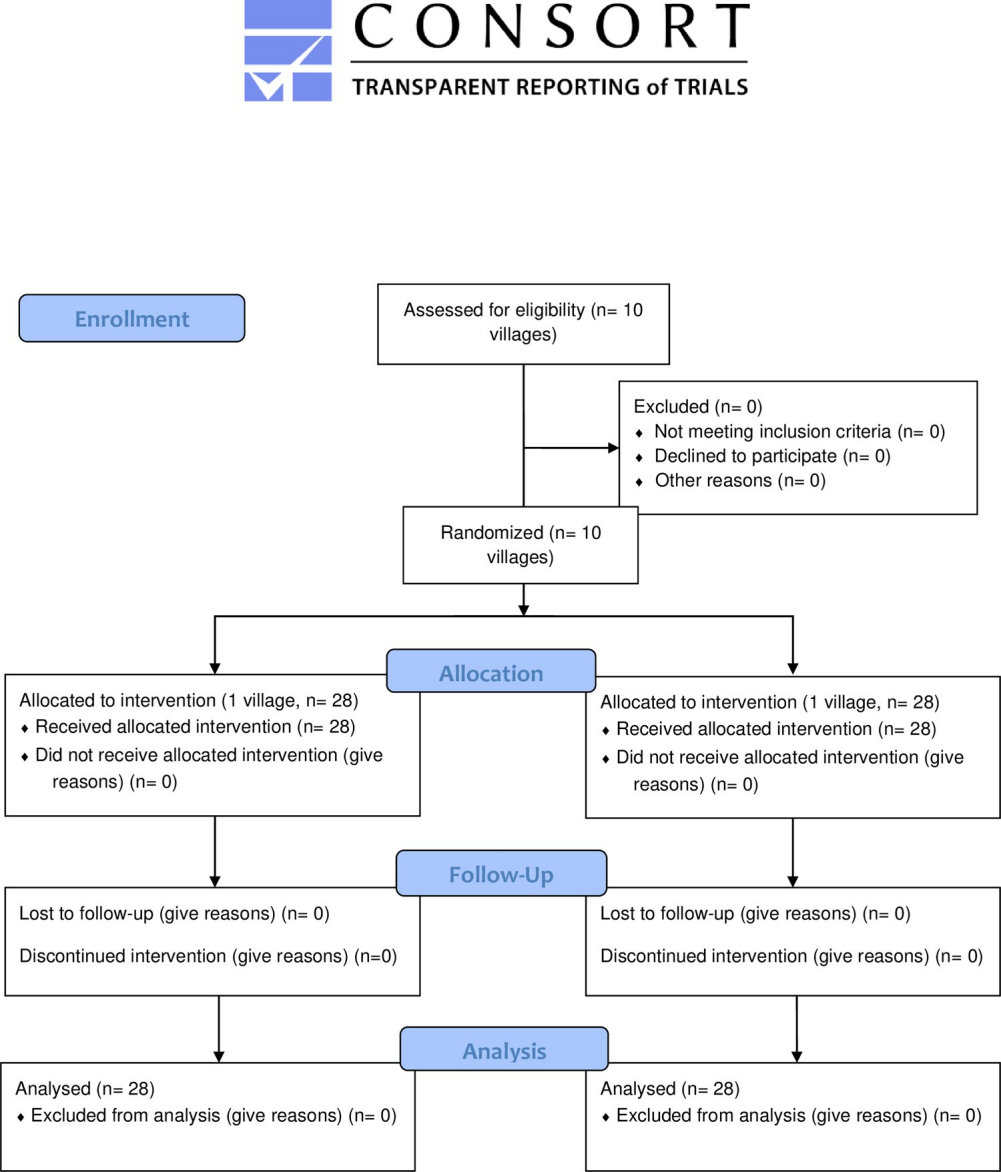
CONSORT 2010 flow diagram containing details of participant enrolment and progress through the RCT.

The study was approved by the Research Ethics Committee of Khon Kaen University, Thailand on 1 March 2019 (HE 612355), and was registered retrospectively with the Clinical Trials Registry of Thailand on 9 July 2020 (TCTR20200709001) consistent with procedures for RCTs in which no medications are prescribed. The authors confirm that all ongoing and related trials for this intervention are registered. Recruitment took place between 10 March and 31 October 2019 and all participants gave fully informed written consent of their willingness to participate.The CONSORT 2010 Flow Diagram containing details of participant enrolment and progress through the RCT is displayed in Fig 1.

### Participants

Inclusion criteria were women aged 60 to 80 years living in Khon Kaen who discussed their participation with their community doctor. Although not used as a screening procedure the blood pressure of each participant was measured before the study commenced. In particular, blood pressure was measured by using a Omron HEM-7124 blood pressure monitor (Omron Healthcare Company Limited, Kyoto, Japan). The whole measurement process is almost automatic. The cuff is placed around the bare arm 1 to 2 cm above the elbow joint. The device is then turned on and the cuff inflates automatically to start the measurement, deflates when the measurement has been taken and the blood pressure reading appears automatically on the screen. Mean Arterial Pressure (MAP) is the average pressure in a person’s blood vessels during the cardiac cycle and was estimated by using a formula in which the lower (diastolic) pressure is doubled and added to the higher (systolic) pressure and the composite sum is then divided by three. This calculation accounts for the fact that the period of diastole lasts approximately twice as long as the period of systole. Low MAP can cause inadequate blood flow to organs, syncope and shock and high MAP contributes to increased oxygen demand by the heart, ventricular remodeling, vascular injury, organ damage and stroke. A small number of subjects had elevated blood pressure or high blood pressure of stage 1 or 2 as defined by the American Heart Association but were still encouraged to participate by their doctor on the basis that the low intensity physical exercise could be beneficial for their health and were appropriately monitored, including regular measurement of blood pressure, throughout the study.

Participants were also required to have good mental faculties and ability to communicate with the investigators in Thai or another language, normal recognition of people, time and place as tested using the Thai version of the Mini-Mental State Examination. Exclusion criteria were serious joint pain, disease or injury that contra-indicated performance of SSG exercise, history of diseases affecting movement, history of major injury due to a fall in the previous year, smoking or alcohol consumption. Additionally, participants should not have regularly performed physical exercise during the past 6 months.

Recruitment of participants proceeded smoothly. This was due to their being great enthusiasm to participate, not only by members of the Exercise group but also by members of the Control group who knew that they would be offered the SSG training, albeit without recording of outcome measures, later in the study. Entry to the study was on a first come first served basis in each group and, provided that the subject who had signed up met the relevant inclusion and exclusion criteria, they were enrolled. The target of 28 participants in each group was achieved within 2 weeks of the invitation being given to the Community and there were no dropouts.

### SSG training program

The lead investigator is a Professionally Certified SSG Instructor and taught the SSG exercise to two co-investigators who each led groups of 14 women into which the Exercise Group was divided for convenient supervision of the SSG training. The two group leaders acted as manager of their respective groups during the study period. During the two months that the Exercise Group underwent SSG training, the women in the Control Group continued their normal life and after the experiment was completed, as had been offered at the time of recruitment, received the same SSG training program as the Control Group but without any measurements being recorded.

When performing SSG, and for the measurements of Posture, Flexibility and Gait, participants wore loose, comfortable sportswear and flat shoes. The 8 week training program comprised three SSG sessions per week, each lasting 40 minutes [25]. A detailed demonstration of SSG was given and any questions that the participants had were addressed. All relevant information was also distributed to the participants in written documents. Throughout the eight week SSG training program, the lead investigator, the two group leaders and seven volunteers led the 28 women of the Exercise Group in performing SSG together. They organized and supported the SSG training sessions, checked that the SSG exercise was performed safely and correctly and recorded the outcome measures to be described below. SSG is a low-intensity exercise and heart rate should be increased by no more than 40–50%. To help ensure this was the case, metronomes and music were played to control the rhythm of the exercise.

**Fig 2:**
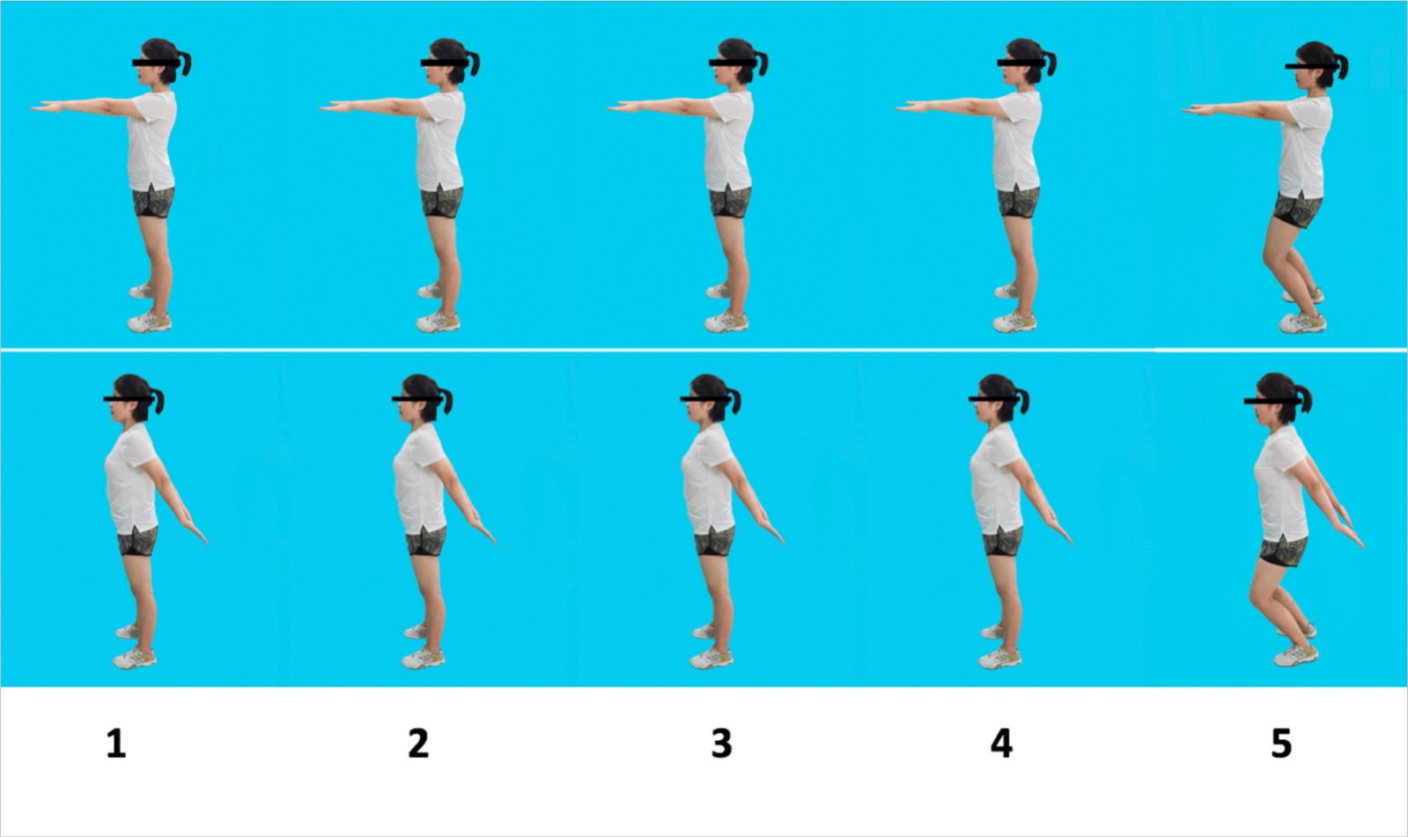
Illustration of Shuai Shou Gong (SSG) exercise. For the first four arm swings the participant maintains their full height and on the fifth swing bends at the knees, once in synchrony with the backward swing and a second time in synchrony with the forward swing. This same cycle of five arm swings is repeated throughout the period for which the exercise is performed.

Each SSG training session was divided into three parts comprising Warm-up, SSG exercise and Cool-down. The 5 minute Warm-up included 30 second intervals of each of the following, raising the neck up and down, shoulder circles, cross arm swings, forward arm swings, wrist circles, trunk twists, knee bends and ankle circles. Next, the SSG exercise (Fig 2) was performed for 30 minutes. The participant stood with their feet shoulder-width apart with their arms hanging comfortably at their side. They then raised their arms out in front to shoulder-height with comfortably straight fingers and performed repeated cycles of movements each comprising of five arm-swings. For the first four-arm swings, remaining upright and keeping their neck and trunk aligned vertically, the participant swung their arms backward and then forward naturally, breathing out through the nose during the backward-swing and in on the forward swing, according to the preset tempo of the metronome. The fifth swing was however different to the rest, with the participant bending their knees one time in synchrony with the backward swing and then a second time in synchrony with the forward swing. The same cycle of five arm swings was repeated throughout the exercise and participants were instructed to swing their arms rhythmically in time with the metronome and music and in a relaxed manner [26]. To assist the participants in developing fitness and stamina in performing the SSG exercise the metronome and music were set so that participants performed 15 arm swings per minute during the first 2 weeks of SSG training, 20 arm swings per minute during weeks 3 to 4, and 25 arm swings per minute during weeks 5 to 8. There was a two-minute break halfway through the 30 minutes of SSG. The approximately 5-minute Cool-down included 40-second intervals of each of the following, neck side stretch, shoulder and upper back stretch, triceps stretch, back stretch, calf stretch, ankle circles and reach for the stars. The following outcome measures were obtained immediately following the first session of SSG on day 1, and again after 4 and 8 weeks, with the latter two recordings being made one day after the relevant SSG session.

### Outcome measures

#### [1] Measurement of posture

The primary measure of Posture that was assessed is C7 to Wall Distance (C7WD) and was measured using metal calipers and a rigid ruler [27]. The participant stood upright with their back and buttocks against a wall and were instructed to stand relaxed and look straight ahead. The spinous process of the seventh cervical vertebra (C7) was palpated and holding the metal caliper horizontally the investigator made light contact of the tips of the caliper with the position of C7 and the wall, respectively. The distance between the tips of the caliper was then measured by using a ruler [28]. The measurement was repeated three times and the mean value recorded. Measures of C7WD have been reported to show excellent intra-rater repeatability and inter-rater reproducibility [29]. The Standing Height (SH) of participants was also measured using a high precision stadiometer.

#### [2] Measurement of flexibility

The Back Scratch test is part of a protocol designed to test upper body flexibility and range of joint motion [30]. In a standing position, participants placed one hand over the shoulder on the same side and with the palm lying against the back extended the fingers as far down the back as possible. The back of the other hand was placed on the back and the fingers extended upward, to attempt to meet the fingers of the hand reaching down from over the shoulder. The fingers of the two hands were aligned and the distance between the tips of the middle fingers of each hand was measured. The measurement was repeated three times for the left (BSL) and right sides (BSR), in random order, and the average value recorded.

Flexibility was also measured using the Chair Sit and Reach (CSR) test [31–33]. For this test participants sat at the front of a chair, with one leg bent at the knee and the other extended forward. On an outbreath, the participant reached forward with the palm of the hand of the opposite side of the body to touch the shin of the outstretched leg as close to the toes as could be managed comfortably. The distance between the tip of the middle finger of the hand touching the shin and the tip of the big toe of the outstretched leg was measured [31], with the test performed in random order for the left (CSRL) and right (CSRR) sides. For each side in random order the measurement was repeated three times and the average value recorded. Jones et al have shown in a study of older women that the CSR has good intra-class test-retest repeatability (R = 0.96) [31].

#### [3] Measurement of gait

For analysis of gait, a measurement was obtained of Timed Up and Go (TUG) which refers to the time taken by the participant to stand up, walk 3 meters, turn around, walk back 3 meters and sit-down [34].

#### [4] Measurement of cognition

The Barthel Activities of Daily Living Index (BADL) was used to measure the functional abilities of participants in performing ten activities of daily living [35]. In addition, the Rosenberg Self-Esteem Scale (RSES) was used to measure the mental state of the participants [36]. For both BADL and RSES Thai versions of the tests were used [37].

### Control group program

The Control Group did not perform any exercise that would act as a comparison to SSG, nor any movement activity that would act as a control to facilitate the study of particular elements of SSG. The rationale for this is that our interest is to investigate the potential benefits of incorporating SSG within normal daily living. Therefore, to control for any effects or variations that might arise on account of, for example, seasonal changes which could influence general activity levels, the same outcome measures were obtained, and questionnaires completed, by the Control Group at the same time intervals and on the same date as for the Exercise Group. Throughout the study investigators also spoke with members of the Control Group to ensure that it would be known if any of the participants adopted significant lifestyle changes that could affect the aims of the study. Motivation for compliance was maintained because, as stated above, after the eight week experiment, the investigators administered the same SSG program to the Control Group.

### Data analysis

In order to assess whether the measurements of the outcome variables were normally distributed the Shapiro–Wilk test was used. This analysis revealed that all outcome measures (i.e. C7WD, SH, BSL, BSR, CSRL, CSRR, TUG, BADL, and BSES) in both the Exercise Group (EG) and the Control Group (CG) were not normally distributed. Non-parametric Multivariate Analysis of Variance (MANOVA) was therefore performed to investigate whether a significant interaction effect existed between group and time for the 8 week SSG training for the set of all outcome measurements and for the four categories of (1) Posture (C7WD and SH), (2) Flexibility (BSL, BSR, CSRL and CSRR), (3) Gait (TUG) and (4) Cognition (BADL and RSES). The particular analysis that was performed used the MANOVA.RM package of R software (R Core Team (2020). R: A language and environment for statistical computing. R Foundation for Statistical Computing, Vienna, Austria. URL https://www.R-project.org/). A Modified ANOVA-Type Statistic (MATS) p-value of < 0.05 was considered for analysis of the set of all variables, since the covariance matrix was singular. Subsequently, a Wald-Type Statistic (WTS) p-value of < 0.05 was considered for the analysis of the four categories as the covariance matrix was not singular.

## Results

### Progress through the trial

Demographic information relating to the participants in the Exercise Group and the Control Group is presented in Table 1. There is good matching between the two groups with no significant differences in average age, height, weight and heart rate of the two groups. A minor, but not clinically significant, difference in blood pressure was observed between the two groups. However, there is no significant difference in the value of MAP between the Exercise group and the Control group and the value in both groups is at the midpoint of the normal range of 70 and 110 mm Hg, providing further evidence of good matching between the Exercise group and the Control group.

**Table 1:** Baseline demographic information (mean ± SD) for participants in the Exercise Group and Control Group.

	Exercise Group (n = 28)	Control Group (n = 28)	*p*-value
Age (years)	68.3±5.6	69.4±4.4	ns
Height (cm)	151.3±5.3	150.9±5.3	ns
Weight (kg)	59.8±9.3	57.8±7.7	ns
Body Mass Index (kg/m^2^)	26.1±3.8	25.1±2.6	ns
Blood Pressure			
Systolic blood pressure (mmHg)	122.1±22.6	134.6±14.4	*P*<0.05*
Diastolic blood pressure (mmHg)	67.6±12.6	66.9±9.1	ns
Mean arterial pressure (mm Hg)	85.8±14.5	89.5± 7.8	ns

*indicates statistically significant difference as analysed using unpaired t-tests (*p*<0.05).

All the women in the Exercise Group completed the SSG training, no participant became unwell during any of the SSG training sessions and no adverse events were recorded. SSG has been developed as an exercise that is especially suited to being performed by older adults. Without any exception the participants enjoyed performing the gentle, rhythmic, non-strenuous, non-competitive, physical activity together and with no need to over reach, risk take or put undue pressure on themselves to perform. Furthermore, as described above, a health check had been performed with a doctor present for all participants at the start of the training and on each occasion that the group met a sizable team was in attendance to check and ensure that, participants always remained comfortable to take it easy or take a rest. There were also no drop-outs in the Control Group. All subjects completed all assessments and therefore a complete dataset could be analyzed referring to a total of 56 participants.

### Results of non-parametric MANOVA analysis

**Fig 3:**
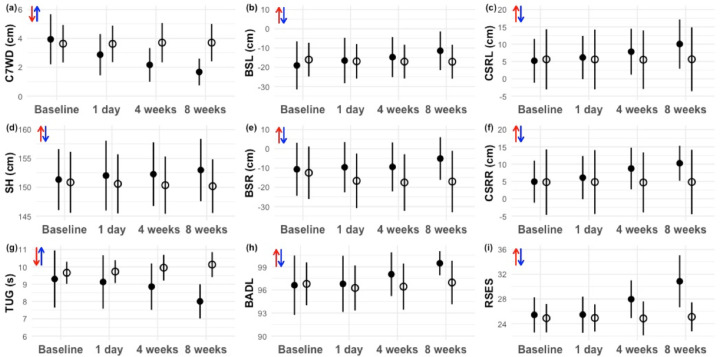
Plots of changes in the outcome measures for subjects in the Exercise Group (filled circles) and Control Group (open circles) during the 8-week SSG training program. Error bars indicate mean ± SD with units indicated in parentheses. The Posture measures C7WD and SH are plotted in (a) and (d), respectively. The Flexibility measures BSL and BSR, are plotted in (b) and (e), and CSRL and CSRR are plotted in (c) and (f), respectively. The Gait measure TUG is plotted in (g). The Cognition measures BADL and RSES are plotted in (h) and (i), respectively. The direction of the short red arrow denotes the direction of change that is predicted to represent a beneficial effect and the direction of the short blue arrow denotes a deterioration.

Results for the outcome measures are presented in Table 2 and plotted in Fig 3, which shows the changes recorded at the four time points of prior to, one day after, four weeks after, and at the end of eight weeks of SSG training. The 8 week SSG training course produced a significant interaction between group and time for the combined set of all outcome measures (C7WD, SH, BSL, BSR, CSRL, CSRR, TUG, BADL, and BSES) (MATS p-value < 0.001) and the four categories of posture, flexibility, gait, and cognition (all WTS p-values < 0.05). Posture improved significantly in the Exercise Group compared to the Control Group over the SSG study period, as reflected by a decrease in C7WD and increase in SH. Flexibility also showed significant improvements with increases in BSL, BSR, CSRR, and CSRL consistent with predictions. With regard to gait, TUG became significantly shorter for participants in the Exercise Group compared to the Control Group. In addition, with regard to cognition there were significant improvements in both BSES and BADL scores in the Exercise Group compared to the Control Group.

**Table 2:** Nine outcome measures recorded at four time points, namely prior to, during (i.e. after 1 day and after 4 weeks) and at the end of (i.e. after 8 weeks) of SSG training in the Exercise Group (EG), and at corresponding times in the Control Group (CG). The measures include C7WD, SH, BSL, BSR, CSRL, CSRR, TUG, BADL and BSES. Values are shown as mean and SD with units indicated in parentheses. BADL and BSES indicate score values for the relevant questionnaire.

**Outcome**	**Group (n = 28)**	**Baseline**	**1 day**	**4 weeks**	**8 weeks**
**C7WD (cm)**	**EG**	3.94 ± 1.74	2.87 ± 1.43	2.16 ± 1.17	1.68 ± 0.93
	**CG**	3.63 ± 1.30	3.62 ± 1.27	3.71 ± 1.36	3.71 ± 1.30
**SH (cm)**	**EG**	151 ± 5.29	152 ± 6.04	152 ± 5.52	153 ± 5.40
	**CG**	151 ± 5.30	151 ± 5.13	150 ± 4.96	150 ± 4.66
**BSL (cm)**	**EG**	-19.0 ± 12.5	-16.5 ± 11.8	-14.8 ± 10.4	-11.4 ± 10.1
	**CG**	-16.0 ± 8.78	-16.9 ± 8.97	-17.1 ± 8.79	-17.1 ± 8.83
**BSR (cm)**	**EG**	-10.7 ± 13.8	-9.58 ± 13.1	-9.42 ± 12.7	-5.08 ± 11.1
	**CG**	-12.5 ± 13.7	-16.7 ± 14.2	-17.5 ± 14.7	-17.0 ± 16.0
**CSRL (cm)**	**EG**	5.20 ± 6.36	6.15 ± 6.25	7.83 ± 6.65	10.0 ± 7.11
	**CG**	5.62 ± 8.70	5.59 ± 8.64	5.51 ± 8.46	5.64 ± 9.22
**CSRR (cm)**	**EG**	4.95 ± 6.10	6.11 ± 6.25	8.75 ± 6.03	10.3 ± 5.06
	**CG**	4.80 ± 9.45	4.84 ± 9.22	4.71 ± 8.68	4.85 ± 9.30
**TUG (s)**	**EG**	9.30 ± 1.65	9.12 ± 1.54	8.86 ± 1.34	8.00 ± 0.99
	**CG**	9.66 ± 0.64	9.72 ± 0.66	9.95 ± 0.74	10.1 ± 0.72
**BADL**	**EG**	96.6 ± 3.86	96.8 ± 3.66	98.0 ± 2.84	99.5 ± 1.58
	**CG**	96.8 ± 2.79	96.2 ± 2.93	96.4 ± 3.00	97.0 ± 2.84
**BSES**	**EG**	25.4 ± 2.85	25.5 ± 2.92	28.0 ± 3.06	30.9 ± 4.20
	**CG**	24.9 ± 2.30	24.9 ± 2.21	24.9 ± 2.74	25.1 ± 2.35

### Time of onset of significant changes in outcome measures in the treatment group

No significant interaction effect between time and group was found after either one session or four weeks later for all four categories, namely Posture, Flexibility, Gait and Cognition (all WTS p > 0.05) with significant effects only arising after eight weeks (all WTS p < 0.05). Although alterations were shown to be gradual over time the minimum period required to show significant benefit from performing SSG training was 8 weeks.

### Changes in outcome measures in the Control Group (CG)

No significant changes in posture, flexibility or cognition were identified in the Control Group over the measurement period (WTS p > 0.05). However, the value of TUG for participants in the Control Group showed a significant deterioration by increasing measurement values over the 8 weeks (WTS p = 0.003). Thus, whereas a significant improvement in gait was produced by performing SSG training, there was actually a significant decline in gait performance over the 8 week period if no training was conducted.

## Discussion

The main findings of the study are that an 8 week course of training in SSG produced a significant interaction between group and time for the combined set of all outcome measurements (C7WD, SH, BSL, BSR, CSRL, CSRR, TUG, BADL, and BSES) (Modified ANOVA-Type Statistic (MATS) p-value < 0.001) and for the four categories of posture, flexibility, gait, and cognition (all Wald-Type Statistic (WTS) p-values < 0.05) and in all cases the changes in the Exercise Group were in the direction predicted as being beneficial. No significant interaction effect between time and group was found after either one session or four weeks for any of the categories (all WTS p > 0.05) with significant effects only arising after eight weeks (all WTS p < 0.05). Thus although beneficial changes occurred over time the minimum period required to produce statistically significant benefit from performing SSG training was 8 weeks. For the Control Group no significant changes were identified for posture, flexibility and cognition, however a significant deterioration was observed in gait with TUG increasing significantly over 8 weeks (WTS p = 0.003).

The significant reduction in C7WD and increase in SH is consistent with a reduction in forward head position of participants after eight weeks of SSG training. Forward head position increases as a result of aging, osteoporosis and disability [38]. Many exercise programs such as stretching, balance, strength, endurance training, Pilates, elastic band resistance training, training of the cranio-cervical flexor muscles and endurance-strengthening of the cervical flexor muscles are also likely to have beneficial effects on posture such as have been reported in the present study [39–43]. However, SSG has the particular advantage of being simple, easy and enjoyable to perform in the community, mobilizes many important joints, including shoulder, elbow, hip, knee, and ankle, and is expected to increase joint stability and the mitigation of undue loads on joints and soft tissues, which will assist in the maintenance of good posture [44].

The results of the Back Scratch test (BSL and BSR) and Chair Sit and Reach test (CSRL and CSRR) showed that there was a significant improvement in flexibility in the Exercise Group compared to the Control Group and, as for posture, all flexibility measures exhibited gradual changes in the predicted direction during the period of training (Fig 3). Repeatedly swinging the arms stimulates the nerves, tendons and muscles surrounding the shoulder joint. Furthermore, repeat contractions of latissimus dorsi and gluteus maximus muscles can result in force being transferred via the lumbar fascia to increase rotation of the torso [45].

The 8 week SSG training program also produced a significant improvement in TUG. On the fifth swing of SSG, participants are required to slightly bend their knees and dip down twice, once on the backward swing and again on the forward swing. Thus, during this element of the SSG exercise there is a coordinated movement involving both hamstrings and quadriceps which is likely to increase the ease of performing sit-to-stand and maintenance of body stability in TUG [46–48]. The reduction in TUG can also be interpreted to reflect an increase in Gait Speed (GS) and which is consistent with the report by Shigematsu et al. [49], that a combination of aerobic dance and balance exercises produced improvements in lower limb muscle strength, one-leg balance, functional extension and GS in adults. All of the major muscle groups that are involved during the gait cycle, namely gluteus maximus, iliopsoas, hamstrings, quadriceps, triceps surae, tibialis anterior are conditioned by the performance of SSG, and although in SSG there is no actual forward movement, as reported by Henwood and Taaffe (2006) [50], obtaining increased muscle strength and body stability can contribute to enhancements of walking and hence GS. In addition, Barnett et al. (2003) [51] reported that combination training may have a beneficial effect on gait and, because SSG combines aerobic fitness and upright balance training with lower limb exercise, SSG can be expected to have a similar effect.

Finally, the results of the questionnaires provided evidence of the wide ranging benefits of SSG relevant to cognition. In particular, the results of the Barthel Activities of Daily Living Index (BADL) questionnaire showed that enhancements had occurred in the ease of performing typical activities of daily living, such as washing, dressing, preparing food, in the Exercise Group compared to the Control Group. This finding is consistent with reports that other exercise, including Qi-Gong [52], Pilates training [53], and aerobic and resistance exercise [54] have been shown to improve the activities of daily living in older adults. In addition, exercise can lead to improvements in mental state and a report that exercise has a positive effect on self esteem, even in the short term [47], is supported by the results of the Rosenberg Self-Esteem Scale (RSES) questionnaire in the present study.

There are two observations relating to the data that were acquired and the findings obtained in the present study that should be discussed. Firstly, there are no missing data for all 56 participants for the measurements that were recorded at baseline and after 4 and 8 weeks of SSG training. Perhaps it is surprising that the database entries are complete and that the need never arose to impute missing values. However, there are two reasons that may account for this. Firstly, the SSG classes that were offered to the Community were extremely popular and there was great enthusiasm to participate and attend all the sessions that were offered. This was probably because SSG is a gentle exercise that is comfortable to perform, is expected to have a positive effect on health and well-being, was offered within a convenient setting at a convenient time, and, not least, was performed in a group setting which reduces inhibitions, and increases engagement and enjoyment. The participants were also interested to learn at the end of the study whether the exercise was proven to be beneficial for the Community. Secondly, the outcome measures were recorded by a dedicated team of young researchers who engaged with the members of the community and always offered gentle encouragement and kind support.

Secondly, for the combined set of all outcome measurements (C7WD, SH, BSL, BSR, CSRL, CSRR, TUG, BADL, and BSES) and for the four categories of posture, flexibility, gait, and cognition an 8 week course of training in SSG produced a significant interaction between group and time and in all cases the changes in the Exercise Group were in the direction predicted as being beneficial. Therefore there is perhaps some concern about the fact that in making measurements and assessments, the investigators were not blinded to whether participants were from the Exercise group or the Control group, and they also knew whether the measurement referred to Baseline, or to after 4 or 8 weeks of SSG training. This potentially creates a situation that is contrary to the principle of equipoise whereby investigators involved in both designing and carrying out clinical trials should be free of any preferences. However, complete blinding would have been very impractical in the present study and great care has been taken to avoid the potential of biases being introduced. There are several facts that suggest that the results that have been obtained are reliable. In particular, the outcome variables were objectively measured using standardised procedures involving the use of accurate measuring devices, rulers and a stop watch, the observer could not see a record of previous measurements when recording the potential change that had occurred after 4 weeks, and then between 4 weeks and 8 weeks. Furthermore, none of the outcome variables that have been studied can be described as exploratory or speculative, and it was not unexpected that improvements in posture, flexibility and gait would occur together in a person who is functioning better as a result of exercising.

There are also some more general limitations to be discussed. Firstly, there is an element of subjectivity in the measurement of some of the outcome variables. For example, when recording standing height and C7WD as measures of posture it is possible, consciously or perhaps unwittingly, to encourage a participant to stand tall. Provided the chair and the distance to be covered is the same on each occasion, the situation is less subjective when a stop watch is used for TUG measurements. However, ultimately, it is desirable for a study such as the present one to be performed using entirely objective measurement systems such as a 3D motion analysis system and Electromyography. Secondly, while there is no evidence of any systematic errors [55] being present in the measurements, these could have been introduced by, for example, recording TUG using a different type of chair, or different distance measurement, at say 4 or 8 weeks. Suzuki et al. (2016) [56] have described a method whereby, if they are recognised, treatment of systematic errors can be included in the analysis. Thirdly, only two Communities were studied, one which served as the Exercise group and one as the Control group. In future work it is important to study whether the same benefits of SSG training are replicated in other Communities, and consideration should be given to comparing the SSG training groups with groups performing an alternative exercise. One advantage of studies of the effect of exercise training programmes is that there is only a small chance of transference of effects between clusters, compared to if, for example, recommendations regarding medication, dietary changes or behaviour modification were shared between members of the two groups. Fourthly, only female participants were recruited, and it will be interesting to repeat the study in a male population and also to study both women and men with a wide range of ages.

In addition to the above consideration of potential limitations of the study, the cluster based sampling scheme should also be discussed further. If the Exercise group had been recruited through contacting, for example, a Yoga Club and the Control group by contacting a Chess Club it is likely that participants from the Yoga Club may be physically more flexible at the start of the study and perhaps readily become more flexible as the training progressed so confounding an investigation of the benefits of SSG. More subtly, a similar confound could have been introduced if perhaps one of the Communities was located near to a transport route so that residents were frequently employed in office work in the city whereas the other Community was in a more remote location and residents worked locally and were engaged in farming and physical work. Hess et al. (2015) [57] have described how in designing a cluster based study the number of participants should be increased to take account of the above considerations and ensure appropriate significance levels and power. In retrospect, this so-called design effect could have been considered in performing the power calculation for the present study. However, the present study, has revealed significant benefits of SSG training in terms of Posture, Flexibility and Gait and what is important now is to investigate whether the benefits of SSG can be reproduced in other Communities and in the population at large. In view of SSG being a group activity, cluster sampling will remain a key aspect of the design of future experiments. Methods for calculating the number of clusters, and the number of participants to be recruited per cluster, have been developed [58, 59] and are discussed by Hemming et al. (2011 and 2017) [60, 61].

## Conclusion

An 8 week program of Shuai Shou Gong (SSG) training delivered over twenty four sessions to 28 older women from the same community, and to a Control Group, produced a significant interaction between group and time for the combined set of all outcome measurements (C7WD, SH, BSL, BSR, CSRL, CSRR, TUG, BADL, and BSES) (MATS p-value < 0.001) and for the four categories of posture, flexibility, gait, and cognition (all WTS p-values < 0.05). In all cases the changes in the Exercise Group were in the direction predicted to be beneficial. Taken together, these findings demonstrate that the gentle, rhythmic, whole body sequence of movements of SSG may be readily learned and enjoyed in a group setting by older adults and improves general health and well-being. In future studies, it will be interesting to further investigate the mechanisms by which the ancient practice of SSG has produced the benefits documented in this RCT.

## Supporting information

S1 File CONSORT checklist file. (DOC)

S2 File Ethics committee approved certificate file. (PDF)

S3 File Ethics committee approved trial study protocol file. (PDF)

S4 File CONSORT 2010 flow diagram. (DOC)

S5 File (DOCX)

S6 File (DOCX)

S1 Data (XLSX)

S2 Data (DOCX)
