# Supplementary material for: The benefits of Shuai Shou Gong (SSG) demonstrated in a Randomised Control Trial (RCT) study of older adults in two communities in Thailand
Source: PLoS One. 2023 May 25;18(5):e0282405. doi: 10.1371/journal.pone.0282405 (PMC10212083; doi:10.1371/journal.pone.0282405)
Supplement: S2 File — (PDF) [file pone.0282405.s002.pdf]

KHON KAEN UNIVERSITY

This is to certify that

The Project Entitled: Arm Swing Exercise for posture and gait development on elderly females

Principle Investigator: Miss Xiao Zhen
Faculty of Associated Medical Sciences, Khon Kaen University

Co-Investigators: Associate Professor Wichai Eungpinichpong
Faculty of Associated Medical Sciences, Khon Kaen University
Associate Professor Dr. Uraiwan Chatchawan
Faculty of Associated Medical Sciences, Khon Kaen University
Associate Dr. Xingze Wang
Research Center on Physical Education and Health Education in Gannan Normal University, Ganzhou, China
Miss Yu Jiao

Documents Acceptance:

1. KKUEC Application form, version 1.1, dated 20 February 2019
2. Clinical Trial Protocol, version 1.1, dated 20 February 2019
3. Information Sheet, version 1.1, dated 20 February 2019
4. Informed Consent Form, version 1.1, dated 20 February 2019
5. Study Recruitment Flyer, version 1.1, dated 20 February 2019
6. Research Instrument, version 1.1, dated 19 February 2019
7. Investigator's Curriculum Vitae

Record No. 4.2.02: 01/2019
Reference No. HE612355

have been reviewed by the Khon Kaen University Ethics Committee for Human Research based on the Declaration of Helsinki and the ICH Good Clinical Practice Guidelines. **Please submit the renewal report within January 06, 2020**

Date of Approval: 01 March 2019

Date of Expiration: 06 January 2020

(Professor Sastri Saowakontha,M.D.)

Chairman of Panel 2

The Khon Kaen University Ethics Committee for Human Research

Record No. 4.2.02: 01/2019

Reference No. HE612355

Office of The Khon Kaen University Ethics Committee in human research
Office of President building 2 Floor 2 nd
Khon Kaen University, 40002 Thailand
Tel.: +66-43-203331, 42942 Fax: +66-43-203331
Office of The Khon Kaen University Ethics Committee in human research (Sub office)
Room 5317, 3rd Floor Wadwichakarn Building, Faculty of Medicine, Khon Kaen University
Mobile 089-7141913 Tel. 67133 – 4

Institutional Review Board Number: IRB00008614
Federal wide Assurance; FWA00003418
