# Supplementary material for: The benefits of Shuai Shou Gong (SSG) demonstrated in a Randomised Control Trial (RCT) study of older adults in two communities in Thailand
Source: PLoS One. 2023 May 25;18(5):e0282405. doi: 10.1371/journal.pone.0282405 (PMC10212083; doi:10.1371/journal.pone.0282405)
Supplement: S3 File — (PDF) [file pone.0282405.s003.pdf]

To Chairman of The Khon Kaen University Ethics Committee in human research

I, Xiao Zhen, of faculty of Associated Medical Sciences would like to submit a research proposal entitled ผลของการออกกำลังกายแบบแกว่งแขนต่อการปรับปรุงท่าทางและการเดินในผู้สูงอายุ (Arm Swing Exercise for posture and gait development on elderly persons) for approval of ethics in human research. I have attached the following documents for your considerations:

1. Submission fee receipt
2. One original and two copies of KKUEC's Submission form for Clinical trial / Experimental study (specify version and dated).
3. Three copies of research proposals (specify version and dated).
4. Three copies of Information Sheet for research subjects (specify version and dated). (See example in KKUEC's website)
5. Three copies of Informed Consent Form for research subjects (specify version and dated). (See example in KKUEC's website) or Written Consent Waive Form (See example in KKUEC's website)
6. Three copies each of Principal investigator's and co- investigator's curriculum vita in Thai or English, and certificate of participation in a workshop for ethics in human research
7. Three copies of research tools (specify version and dated).
8. Three copies of submit form for Thesis/Independent Study Proposal graduate study, Khon Kaen University (GS23) (In case of graduate students)
9. One set of CD/DVD containing data of number 2-8

Thank you for your kind considerations.

KKUEC's Submission Form for Ethics in Human Research for Clinical trials/Experimental studies

The applicant must submit details on related topics (respond to every item, if an item is not applicable to the submitted project, write 'not applicable'. Do not leave any item blank).

1. Research title: ผลของการออกกำลังกายแบบแกว่งแขนต่อการปรับปรุงท่าทางและการเดินในผู้สูงอายุ (Arm Swing Exercise for posture and gait development on elderly persons)

2. Principal investigator and affiliation: คณะเทคนิคการแพทย์ (Faculty of Associated Medical Sciences) Phone number 06-5561-3286. E-mail 526111931@qq.com

3. Co- investigator(s) and affiliation(s): Associate Professor Wichai Eungpinichpong, PhD, PT Division of Physical Therapy, Faculty of Associated Medical Sciences, Khon Kaen University รศ.ดร.วิชัย อึ้งพินิจพงศ์ ภาควิชากายภาพบำบัด คณะเทคนิคการแพทย์ มหาวิทยาลัยขอนแก่น

4. Significance of problems to be studied (executive summary)

While Arm Swing Exercise has been found to increase some range of motion of shoulder joint and pulmonary functions (Tunkamnerdthai et al., 2015), it has some limitations such as being too boring and could cause shoulder pain if it is not done properly or done too long. It has mild to moderate effects on the cardiovascular system. However, it might be a good exercise for improving posture and gait because swinging the arm during exercise requires erect standing and postural control.

ASE exercise is a Qigong traditional Chinese exercise characterized by simple, slow, relaxed movements which are easy to learn. Theoretically, it should be an ideal exercise for improving body balance, posture and gait. However, its effect on posture and gait has not been explored. At present, there is no research on the physiological mechanism effect of ASE exercise on the posture and gait of the elderly. This study uses ASE exercise as an intervention training method.

5. Objectives (Write clearly)

The objective is to investigate the effects of ASE has on physiological (Barthel Activities of Daily Living Index, Heart Rate, Blood Pressure, Heart Rate Variability), psychological (Self-esteem), posture (Occiput-To-Wall distance, Standing Height, Back Scratch, Sit-and-Reach) and gait (Spatio-temporal-Parameters, Time Up and Go), biomarkers after exercise and to verify the effect on gait and posture in elderly people after exercise.

6. Concrete benefits of the project once completed:

The random controlled trial is an appropriate designed to verify the effects of this exercise in elderly people. This study goal is to promote the elderly's posture, gait, physical health, and quality of life through the ASE.

7. Types of studies and research design:

☑ a. Treatment study (Specify)............................

☐ b. Diagnostic study (Specify)............................

☐ c. Epidemiological study (Specify)............................

☐ d. Descriptive study (Specify)............................

☐ e. Others (Specify)............................

8. Background of study in humans

a. Brief research background with references

With aging, there are obvious physiological and psychological changes. The following physiological changes include the following aspects: appearance of osteoporosis, strength decline, cardiovascular diseases, sensory disorder, decline of cognitive function, and balance mobility performance decreased (Challenge, 1990). Psychological changes include the following aspects: loneliness, dependency, emotionally unstable, and depression. Psychological and physiological changes have a huge impact on the posture and gait of the elderly. The following changes are seen in the posture of the elderly: forward head, thoracic kyphosis, lumbar lordosis, and hip flexion. The following changes in gait are commonly found: slower walking speed, shorter step length, step width, and stride length (Burnfield, 2010). Bad posture and gait seriously affect health and living quality of the elderly. Aerobic exercise, muscle strengthening, endurance exercise and flexibility exercise have been proposed as intervention to improve posture and gait.

The intervention training methods for improving posture and gait of the elderly obtained by literature retrieval include the following: Pilates exercise, stretching exercise, balance exercise, strength exercise, endurance training, elastic band resistance training, combined exercise, training of the craniocervical flexor muscles, and endurance-strengthening of the cervical flexor muscles (Whittle, 2007).

ASE has been used for maintaining good posture as well as wellbeing by Chinese people for long time. Since it is simple to practice and involves many core muscles contraction rhythmically during the swinging of the arms, it may have a role on improving cardiovascular, muscular strength, range of motion, and postural control. Nowadays, the research field has not yet decided which type of intervention training can best promote the posture and gait of the elderly. The current research will use traditional Chinese ASE (Shuai Shou Gong) as an intervention exercise.

Shuai Shou Gong (SSG), a version of Arm Swing Exercise (ASE), is a kind of traditional Chinese QiGong that has been practiced by people for more than one thousand years to maintain physical well-being. Shuai Shou Gong first appeared in the book of Dharma Yi Jin Jing more than a thousand years ago (Kejin, 2014). The Chinese traditional ASE process includes: the participants stand with the two feet parallel, keep body upright, eyes closed, the whole body relaxed, inhale and exhale through the nose, keeping the body natural and relaxed, then open eyes and smile. Hands up and apart about the same width of the shoulders, raise both hands parallel to the ground, keeping fingers straight (MeiMen YiQi Popular, 2018). Their hands swing back and forth naturally and follow the tempo. The ASE consists of five arm-swings, where the action from 1 to 4 is the same. However, on the fifth swing, the participants slightly bend their knees and dip down twice. They swing rhythmically in a relaxed way to form a natural rhythm (MeiMen YiQi Popular, 2018).

The performance of ASE consists of a series of rhythmic arm swinging and body swaying back and forth which improve blood circulation, physical expenditure level, and promotes physical fitness and well-being. It has been classified as a low-intensity exercise, based on approximately 23% of the maximum VO2 and 45% of the maximum heart rate (HR Max) during exercise (N Leelayuwat, 2013). Based on traditional Chinese medicine, ASE may facilitate circulation of Qi, the internal energy (Minghui, 2004) throughout the whole body. Since Qi nourishes the internal organs and helps recovery from illnesses (MeiMen YiQi Popular, 2018), ASE is believed to provide positive effects for health.

Performing ASE quickly and repeatedly stimulates the muscles, improves the transmission rate of nerve transmissions across synapses, improves tonic reflexes that cause the muscles to contract and increase muscular activity (Zhengwei, 2004). ASE is beneficial for increasing muscle strength and

balance stability. Arm swinging is an aerobic exercise, which is helpful for the stimulation of vestibular function (Fleg, 2012) as vestibular function is significantly improved. Training has the most obvious effect on proprioception (Fleg, 2012). Rapid vibration stimulates the receptors in the joints, muscles and ligaments, activates reflection and proprioception circuits, improves the sensory function of upper limbs and promotes neuromuscular recovery (Gertenbach, 2002). ASE is conducive to the accurate coordination of space and time between the sensory center and muscle proprioception (Kai, 2015).

Theoretically, repeatedly swinging the arms stimulates the nerves, tendons and muscles surrounding the shoulder joint. When the arm swings, the latissimus dorsi muscle and the gluteus maximus are connected by the superficial layer of the lumbar fascia which can transfer the force and rotate the torso (Pool-Goudzwaard, 2013). Because there are many Qi channels connected to different organs that end on the hand, the nerve endings through the swinging arm movement increases the circulation of these channels (Jwing-Ming Y., 2002).

In previous studies, ASE has shown a protective effect on vascular complications by improving blood glucose control and improving oxidative stress through exercise (Naruemon Leelayuwat et al., 2008). ASE increases oxygen consumption and exercise capacity in normal-weight and overweight young people (Naruemon Leelayuwat et al., 2008). The ASE shows improvement of pulmonary functions in type 2 diabetes mellitus (T2DM) patients after ASE training (Gleeson, Nieman, & Pedersen, 2004). Shoulder periarthritis patients through 45 days of exercises can effectively improve the symptoms of shoulder periarthritis, relieve shoulder pain, and expand the range of motion of the shoulder (Changxin Wang, 2000).

When doing Qigong exercises, the cerebral cortex encounters a low mental load and low mental energy expenditure. Qigong exercise should be done under a static state, so that alpha wave enhancement, such that enhancement and diffusion of active inhibition in the cerebral cortex can occur. This changes the perceptual and the autonomic nervous systems that regulates visceral activity changes, which show decreased sympathetic excitability activity (Xu bing, 2000).

Good posture contributes to efficient walking and reflects wellbeing. Elderly people tend to have problems with posture and gait as they get older and frail, which leads to decreased activities in daily living and makes them prone to falling and disabilities (Salzman, 2010). Because the proportion of the elderly in most countries in the world is increasing, it is very important to study all aspects of gait and posture in order to prevent falls and facilitate independence in the elderly. For the elderly person's biomechanics, Balance requires the coordination of multiple sensory system, which including the visual systems, vestibular, and somatosensory (Gribble, Hertel, Denegar, & Buckley, 2004).

There are very few research reports on how good posture of the trunk and gait can be enhanced by ASE and consequently develop physical balance. Functional exercise is a kind of therapy, which can promote improved motor function. The therapy involves muscle strength exercise, motion of joint activity, and balance and coordination function exercise. This study will use ASE exercises to conduct intervention experiments. In the group study, the experimental group will do ASE exercises for the elderly through experimental intervention, and the control group will not perform the exercises. Before and after the experiment intervention, the evaluation parameters will be measured and evaluated, to explore whether ASE is beneficial for the elderly to obtain optimized positions, and whether they can help repair posture and gait. The effects of ASE exercise on posture and gait will be investigated by the test of the parameters before, during and after the intervention. Through data analysis, this study will try to determine whether ASE Exercise will help improve the posture and gait of the elderly.

References

Burnfield, M. (2010). Gait analysis: normal and pathological function. (Vol. 9). Journal of Sports Science and Medicine. 353.

Challenge, T. H. (1990). The Health-Care Challenge.

Changxin wang, zhou guangfu. (2000). shuai shou gong threapy Treatment of scapulohumeral periarthritis. Journal of Avant-Garde Medicine, 17(6), 356–357.

Fleg, J. L. (2012). Aerobic exercise in the elderly: A key to successful aging. Discovery Medicine, 13(70), 2012.

Gertenbach, H. J. (2002). Gertenbach, H. J. (2002). The influence of proprioceptive training on the functional balance of older adults, Doctoral dissertation, Stellenbosch: University of Stellenbosch.

Gleeson, M., Nieman, D. C., & Pedersen, B. K. (2004). Exercise, nutrition and immune function. Journal of Sports Sciences, 22(1), 115–125.

Gribble, P. A., Hertel, J., Denegar, C. R., & Buckley, W. E. (2004). The Effects of Fatigue and Chronic Ankle Instability on Dynamic Postural Control. Journal of Athletic Training (National Athletic Trainers' Association), 39(4), 321–329.

Jwing-Ming Y. (2002). Swinging the Arms or Bai Bi.

Kai, Z. yun. (2015). Effects of whole body vibration training on muscle strength and balance ability of lower limbs in the elderly, 35(4), 79-86

Kejin, C. (2014). Dharma Yi Jin Jing. Chengdu times press. Chengdu: Chengdu times press. https://doi.org/10.1016/j.ijnurstu.2011.09.009

Leelayuwat, N. (2013). Beneficial Effects of Alternative Exercise in Patients with Diabetes Type II. Retrieved from http://cdn.intechopen.com/pdfs/45373/, InTech-Beneficial effects of alternative exercise in patients with diabetes type

Leelayuwat, N., Tunkumnerdthai, O., Donsom, M., Punyaek, N., Manimanakorn, A., Kukongviriyapan, U., & Kukongviriyapan, V. (2008). An alternative exercise and its beneficial effects on glycaemic control and oxidative stress in subjects with type 2 diabetes. Diabetes research and clinical practice, 82(2), e5-e8.

MeiMen YiQi Popular. (2018). MeiMen YiQi Popular.

Pool-Goudzwaard, A. (2013). Biomechanics of SI joint and the pelvic floor. Journal of Chemical Information and Modeling (Vol. 53).

Salzman, B. (2010). Gait and balance disorders in older adults. American Family Physician, 82(1), 61–68.

Whittle, M. W. (2007). Gait anlysis an introduction (4th ed.). Heidi Harrison.

Xu bing, wang xiaodao. (2000). Psychosomatic medicine- Basic and clinical, 235–237.

Zhengwei, G. (2004). Physical education teaching theory. Beijing: Beijing sports university press.

b. Does this study have been conducted in humans before?

Yes

c. If this study has been conducted in humans, explain why it needs to be replicated?

Previous studies have been conducted in humans, However, the effect of ASE on the posture and gait of the elderly has not been verified. This study will try to determine whether ASE will help improve the posture and gait of the elderly.

d. If this study has not been conducted in humans, has it been fully studied in animals?

Not related

9. Population and research subjects

a. Number of research subjects

56

b. How is the number calculated (show statistical formula and calculation method)?

$$n/\text{group} = \frac{2\sigma^2(z_\alpha + z_\beta)^2\,(1-\rho^2)}{(\mu 1-\mu 2)^2}$$

$$\text{Whereas } \sigma^2 = \frac{(n1-1)S1^2 + (n2-1)\ \ S2^2}{n1+n2-2}$$

- ☐ n =sample size;
- ☐ Z_α = standard normal deviate for α, usually set with α = 0.05, $\alpha/2$ = 0.025,

$Z_{0.05}$= 1.645, $Z_{0.025}$= 1.96;

Z_β = standard normal deviate for β, which usually is set to 80% or 90%, β=0.2, β=0.1 respectively, $Z_{0.2}$= 0.842, $Z_{0.1}$= 1.282;

- ☐ S1 = standard deviation of the post-test score in experimental group (S1 = 1.93);
- ☐ S2 = standard deviation of the post-test score of control group (S1 = 2.28);
- ☐ ρ= correlation coefficient between pre and post test score of each outcome measure (Occiput-to-wall distance ρ =0.9);
- ☐ μ_1= mean of outcome measure after experimental group (Occiput-to-wall distance μ_1 = 6);
- ☐ μ_2= mean of outcome measure after control group (Occiput-to-wall distance μ_2 = 7.88);
- ☐ $\mu_1 - \mu_2$ = mean difference between two groups;
- ☐ $n/\text{group} = \frac{2\sigma^2(z_\alpha + z_\beta)^2\,(1-\rho^2)}{(\mu 1-\mu 2)^2}$
- ☐ $n = \frac{8.8098 \times 38.25631555856 \times 0.19}{3.5344}$
- ☐ $n = 18.11991875310763$
- ☐ $n \approx 18$

The dropout rate is set at 20% because the intervention period is 2 months which is relatively long time, the final sample size is calculated as follows:

$$n_{adj} = \frac{n}{(1-R)^2}$$

n: Sample size

R: Rate of dropout

☐ $n_{adj} = \frac{n}{(1-20\%)^2}$

☐ $n = 28.125$

☐ $n \approx 28$

Therefore, the total sample size of this experiment is 56.

c. Inclusion criteria

1) Female, aged between 60- 80 years.
2) Have certain activity ability, certain living capacity.
3) Able to walk independently, or complete the test by walking with equipment
4) No history of related diseases affecting the movement system (Ataxia, Dystonia, Huntington's disease, Parkinson's disease, Tourette Syndrome, Muscular Dystrophy, etc.)
5) No history of major falling injury in the last year
6) No smokers or drinkers.
7) No regular physical exercise for during the past two years.

d. Exclusion criteria

1) Serious joint pain
2) Having some diseases that could be contra-indicate to exercise such as asthma, fever, and injury.
3) Unable to complete data measurement or comply with the requirements of this study.
4) Unable to complete the intervention.

e. Withdrawal of participant criteria means indications that point to dangers that will happen to the volunteers if the research protocol continues.

1) Death.
2) During the intervention, participants withdrew themselves or experienced unexpected conditions, such as serious illness or injury.

f. Termination of study criteria e.g. numerous cases of adverse side effects, or after a period of research study it is found that the study cannot proof the expected efficacy. If this is not appicable, please state "none".

None

g. Are healthy subjects included in the study?

Yes

h. Are the following vulnerable subjects (those who cannot make critical decisions) included in the study?

☐ No

☑ Yes, they are

- ○ Infants, children
- ○ Pregnant women
- ☑ Elderly
- ○ Patients with chronic diseases
- ○ People who cannot give consent by themselves
- ○ People with disability

- Prisoners, alien laborers, in some cases people who are socially disadvantaged, students, and minorities
- Others (Specify)..

If there are vulnerable subjects, please state reasons why this group of subjects must be included in the study. Please also suggest how you plan to protect these vulnerable subjects.

-

i. Method(s) used in getting access to the target population and persuading them to join the project (e.g. advertisement, ads in printed media, radio commercials, or asking for co-operations from the doctor who treats the patients)

Take Advertisement in public to persuade to join the project

j. If there is a monetary or non-monetary reward, please give details and value of the reward.

It will be set up monetary reward in the study. During the course of the experiment, the participants' personal time will be used. It may also delay participants' normal work, thus affecting their salary. Therefore, some economic compensation will be made to the participants.

Details of the value of the reward for the participants (The total price: 134400 Baht)

ASE group (28 person)	The cost of participant's join ASE exercise	per each person (Baht)	Number of times	Total Price each person (Baht)	Total Price (Baht)
		120	24	2880	134400
	Collection data	120	4	480	
Control group (28 person)	Conduct a basic situation survey once a week	120	8	960	
	Collection data	120	4	480	

k. If the study is a randomized controlled trial (RCT), please give details on how the subjects are divided into groups.

This study will use the randomized controlled trial design, which uses two parallel groups, and use a single blind in two elderly communities, Khon Kaen province. First, the elderly people will be assessed for eligibility. If they are eligible, they will be given the baseline evaluation. Two groups will be created, 28 subjects in the ASE group (one community) and 28 in the control group (the other community).

10. Possible effects on the research participants and their compensations

a. Explain if there is any physical, mental, social, and economic risk.

Muscle soreness and fatigue after exercise will occur in the first two weeks of intervention. It is possible that the experimental intervention may lead to the participants' mental stress in the early stage.

b. How has the researcher planned to prevent complications and take care of the participants in case of a complication?

During the intervention process, the elderly should avoid sports injuries. During each intervention exercise, the experimental procedures will be strictly followed: warm-up (the participant will use almost every muscle and joint, from the neck to the ankles in the body), cool-down (Cool-down is an easy exercise done after the exercise, letting the body gradually transition to a resting or near-resting state. Cool down

allows the heart rate to return to its resting rate. It is effective in avoiding injury if performed after exercise). Make sure participants are adequately warmed up and cool-down for each intervention. If there are complications such as sports injuries, physical and psychological treatment will be carried out in time. Ensure participants' physical and mental health.

c. Who will pay for medical care in case of a complication?

Complications still occur if the participants adhere strictly to the protocol, the medical care will be pay by the research grant. If complications occur because the participants don't follow the protocol, the medical care will be borne by the participants themselves.

d. How has the researcher arranged for insurance for damage/injury?

No insurance

11. Treatment method or practice used in the study

a. Explain how the method used in the study is similar to or different from the routine practice.

Treatment training routine more strictly than regular exercise. The treatment training will be carried out in strict accordance with the experimental procedures: warm-up 5 minutes, main training 30 minutes, cool-down 5 minutes.

b. What are the alternative diagnoses or treatments?

Arm Swing Exercise

c. If a placebo is used in the control group, state reasons why this must be used. Give an evaluation of possible risks and benefits.

Not related

12. Does this study involve the test of herbal medicine and natural products?

☑ No. Go to item 14

☐ Yes. Specify if the herbal medicine or drug formulation used has one of the following characteristics.

- A study of medicine in the traditional Thai drug formulation or traditional Thai medicine textbooks that is in accordance with the indication and use of traditional Thai or alternative medicine
- A study of medicine in the traditional Thai drug formulation or traditional Thai medicine textbooks that is in accordance with the indication and use of conventional or alternative medicine
- A study of herbal medicine the use of which is indicated in non-existing conventional medicine, but (the use) can be cited according to the principles of traditional Thai or alternative medicine
- Use of foods or food supplements for health benefits
- A clinical trial study that uses medicine prepared from natural substances in a modern process (pure or semi-pure extracts, and new derivatives)

13. The researcher is to provide the following documents. Make a check mark (√) in the box in front of the documents provided.

☐ If the drug/food/food supplement has been approved by the Food and Drug Administration, attach Package Insert/leaflet.

☐ Document showing indications of use that is in accordance with alternative medicine: targeted disease, dosage, length of time, etc. (Give references of books, traditional Thai drug formulation, or traditional Thai medicine textbooks)

☐ Information on safety in humans, or in laboratory animals if the herbal medicine has not been tested in humans.

☐ Method of herbal drug preparation – is the natural product used the original ancient medicine or is it a coarse extract? Show the preparation procedure.

☐ Scientific reports that support the action of drug under study: study in animals, observations in humans

☐ If this is a study of food or food supplement, provide proofs whether it is generally consumed, a local food, or food that is registered as food for humans.

14. Does this study involve the test of conventional medicine?

☑ No. Go to item 15.

☐ Yes. Give name of the medicine with the following details separately according to types of medicine

1) .... (Indicate usage, amount, and frequency.)

- O The medicine is approved by the Food and Drug Administration (FDA), Ministry of Public Health for the treatment of...(The Package Insert is attached.)
- O The medicine has not been approved by FDA, but it has been studied in humans and Investigator's Brochure Issue no.......... dated.......... Is attached)
- O The medicine has not been approved by FDA nor has it been studied in humans, but is has been studied in animals and the research report or related references are attached.
- O Others. Specify...

15. Does this study involve a test of medical device?

☑ No.

☐ Yes. Give name of the medical device .... with the following details:

a. Details of FDA approval

- O The device is approved by FDA for the treatment of .... The device specifications and operation manual are attached.
- O The device has not been approved by FDA, but it is an adaptation or improvement of a device that is FDA approved. The specifications and operation manual of the new and original device are attached together with information on the technical comparison of the new device with the original one.
- O The device has not been approved by FDA, and it is a newly invented device and has been studied in humans. Related research reports as well as device specifications and operation manual are attached.
- O The device has not been approved by FDA, and it is a new invention that has been studied in animals but not in humans. Related research reports as well as device specifications and operation manual are attached.
- O **Others. Specify.** ....

b. Methods of using the medical device

- O External use. Specify. ....
- O Internal use. Specify. ....

16. Details of examinations involved in the study (Specify examined areas, length of time, and frequency)

a. Specify the examinations with invasive procedure. .... (For example, local or general radiation, general anesthetics, tube or camera insertion)

None

b. Specify the examinations with non-invasive procedure BMI, heart rate and blood pressure (For example x-ray, ECG, EEG, taking blood pressure)

17. What are the specimens that will be taken out of the subjects' body? What is the amount of the specimen, and how often will be the specimen taken?

Not related

18. Subjects' Written or Verbal Informed Consent. Make a check mark (√) in the box.

☑ a. Written consent (Attach the informed consent form and the information sheet.)

☐ b. Verbal consent (Attach the ECKKU-Waiver of Consent form.)

☐ c. Initial verbal consent followed by written consent. (State additional reasons for the issues below and how the written consent will be later secured. Also attach the information sheet for the subjects or representatives.)

1) Does this study involve subjects under critical conditions? Why are these subjects recruited into the study while there are standard treatment procedures?
2) Reason for not being able to secure written consent
3) Does recruiting subjects under critical conditions into the study have a direct benefit for the subjects?
4) Reasons for not being able to conduct this study if permission for a verbal consent is refused.

19. Explain the process of obtaining subject's consent :

19.1) Who is the person who asks for consent? (Consider that the subjects give their consent without undue influence /coercion)

Researcher will ask for consent.

19.2) When are the subjects asked for consent? (Consider that the subjects have an opportunity to ask questions about research and adequate time before making decision)

After explain the participants about the study and objective

19.3) Where does the process of consent take place? (Consider that the place provides privacy and keep the confidentiality of the subjects as well as convenience for the subjects asking questions about becoming a research subject). Please give details.

It will be doing the process of consent in a separate, quiet room Participants are first asked and recorded for basic information. Second, the researchers will be explaining the process, benefits, and associated risks of the experiment to the participants. Finally, participants were left to decide whether or not to participate in the experiment

20. In planning this research protocol, a researcher or biostatistician [ ] has not been consulted. [ ] has been consulted,

Research methodologists:

Name Assoc. Prof. Dr. Wichai Eungpinichpong Signature ..

Name .... Signature ....

Biostatisticians:

Name Assoc.Prof.Dr. Uraiwan Chatchawan Signature ....

Name .... Signature ....

21. Does the research protocol make use of a standard handbook or guidance?

[✓] No [ ] Yes, please identify

[ ] The handbook or guidance has been approved by a professional association or Royal college
Please identify .... (please attach proof)

[ ] The handbook or guidance has received permission to be used by the department/office where it is going to be used. Please identify ....
Name of the person in charge of the department/office ....
Signature ....

[ ] The handbook or guidance has been approved by an expert or experts
Name of expert .... Signature..
Name of expert .... Signature..
Name of expert .... Signature..

22. Is this a multicenter study? If so, please give name(s) of the researchers and participating organization/institution (in Thailand)

None

Please give name(s) of sponsors such as pharmaceutical/chemical companies, *if applicable.*

23. Details of the entire budget for the research project

Item	Total Price (Baht)
Participants compensation	134400
Volunteers compensation	24000
Elderly Leaders compensation	7200
Rental fee of the experimental site	4000
Office material (A4 Paper, Copy fee)	1000
Summary	170600

24. Affiliations of researchers with the companies supporting the research projects such as

☐ Shareholder of the supporting company. State number of shares held.

☐ Owner of medicine or medical device patent, or

☐ Receiving a consultant salary of ....baht per month

☐ Invited to be lecturer from the company, or supported for attending conferences abroad in the past year. Give details...

☐ Others. Specify. ....

☑ No affiliation

25. Research studies in charge

a. At present, how many research projects are you overseeing?

0

b. What is the total number of subjects you are taking care of?

0

c. How do you manage these projects without causing risks to the subjects or other routines?

0

26. Do you have experience in ethics in conducting research studies?

ø The researcher and research team have attended the following training courses for ethics in research studies. Give individual details and proved proofs of attendance.

1) Researcher name: Miss Xiao Zhen Course/training topics: Ethic in human subject (Khon Kaen Hospital) Year taken 2018
2) Researcher name: Assoc. Prof. Dr. Wichai Eungpinichpong Course/training topics: Ethic in human subject for Biomedical research (KKU) Year taken 2017
3) Researcher name:........................Course/training topics:..............................Year taken ....

○ The researcher has not taken the training course, but has planned to develop the potential of the research team according to international standards as follows:

....

27. This research project expects

a. to start collecting data in (month) March (year) 2019 and finish in (month) June, (year) 2019

b. to spend a total of - year(s) 4 month(s)

28. The following documents are attached in requesting the approval from the KKUEC to conduct the proposed research study.

☑ Submission fee receipt

☑ One original and two copies of KKUEC's Submission form for Clinical trial / Experimental study (specify version and dated).

☑ Three copies of research proposals (specify version and dated).

☑ Three copies of Information Sheet for research subjects (specify version and dated). (See example in KKUEC's website),

☑ Three copies of Informed Consent Form for research subjects (specify version and dated). (See example in KKUEC's website) or Written Consent Waive Form (See example in KKUEC's website)

☑ Three copies each of Principal investigator's and co- investigator's curriculum vita in Thai or English, and certificate of participation in a workshop for ethics in human research

☑ Three copies of research tools (specify version and dated).

☑ Three copies of submit form for Thesis/Independent Study Proposal graduate study, Khon Kaen University (GS23) (In case of graduate students)

☑ One set of CD/DVD containing data

I hereby certify that the above information is truthful, and the person filling in the information clearly understand every piece of the information given.

This protocol has been approved by the affiliated organization.
