# Supplementary material for: The benefits of Shuai Shou Gong (SSG) demonstrated in a Randomised Control Trial (RCT) study of older adults in two communities in Thailand
Source: PLoS One. 2023 May 25;18(5):e0282405. doi: 10.1371/journal.pone.0282405 (PMC10212083; doi:10.1371/journal.pone.0282405)
Supplement: S6 File — (DOCX) [file pone.0282405.s006.docx]

# RESEARCH METHODOLOGY

The outline of chapter three: RESEARCH METHODOLOGY

1. Subject

1.1 Inclusion criteria

1.2 Exclusion criteria

1. Sample size determination
2. Study design
3. Randomization
4. Experiment procedures

5.1 Ethical approval

5.2 Procedures

5.3 Management of experiment

5.4 Monitoring of training

1. Exercise protocol
2. Outcome measurements

7.1 Physiological markers

7.1.1 Heart Rate (HR)

7.1.2 Blood Pressure (BP)

7.1.3 Heart Rate Variability (HRV)

7.2 Psychological marker

7.2.1 Rosenberg Self-Esteem Scale (RSE)

7.2.2 Barthel Activities of Daily Living Index (BADL)

7.3 Posture markers

7.3.1 Occiput-Wall Distance (OWD)

7.3.2 Back Scratch (BS)

7.3.3 Chair Sit-and-Reach Test (CSR)

7.3.4 Standing Height (SH)

7.4 Gait markers

7.4.1 Timed up and go (TUG)

7.4.2 Spatio-Temporal Parameter (STP)

1. Statistical analysis

## 1. Subjects

This study used the randomized controlled trial for experiment design based on the research questions. Certain reasons seemed to explain why most researchers often used this design. First, it provided strictly scientific control over the threat to internal effectiveness. Second, it was a versatile design. A prospective, cluster randomized controlled trial was conducted in the elderly community, two parallel groups, in Khon Kaen province, Thailand. females aged 60-80 years, were recruited through a public announcement, from the Khon Kaen elderly community. The researcher selected the participants in the study and also performed detailed physical examinations and collected baseline data on all individuals.

### 1.1 Inclusion criteria

1. Females aged 60- 80 years.
2. Live in Khon Kaen.
3. Have good mental faculties and can communicate in Thai.
4. Normal recognition on person, time, place as tested with MMSE-Thai 2002 and have a score of 14 or more for un-educated persons, 17 or more for primary school educated persons, 22 or more for those who have more than a primary school education.
5. Were able to take care of themselves as indicated by Barthel Activity Daily Living Index, BADL with score 75 or more.
6. Were to walk independently or with equipment.
7. Were to participate in the study.

### 1.2 Exclusion criteria

The participants were excluded if they had one of the following conditions:

1. Serious joint pain.
2. Had a disease or injury that could be contra-indicated to exercise.
3. History of related diseases affecting the movement system
4. History of major injury due to a fall in the last year.
5. Smokers or drinkers.
6. Regular physical exercise during the past 6 months

#### 1.3 Termination criteria

1. Death.
2. During the intervention, participants withdrew themselves or experienced unexpected conditions, such as serious illness or injury.
3. Were not able to complete data measurement or comply with the requirements of this study.
4. Were not able to complete the intervention.

## 2. Sample size determination

The marker of sample size calculation was: The effect of an adapted physical activity program in a group of elderly subjects with flexed posture: a clinical and instrumental assessment. In that study, researchers randomly assigned participants to one of two groups: experimental group performed a physical activity program that was suitable for bending; control group completed a program of non-specific physical activity for elderly persons. Multidimensional clinical evaluations were performed at baseline and after 3 months. The author verified that the adaptive physical activity programs significantly improved posture alignment and flexed posture in older adults.

The researcher used the following formula to calculate the sample size (Borm et al., 2007). The results in the experimental group had µ1 = 6.0, control group had µ2 = 7.88. α = 0.05(Z _0.05_= 1.645) and β power was set to 80%, β=0.2 (Z _0.2_= 0.842) was chosen for sample size calculation.

$n/\mathrm{group}=\frac{2\sigma^{2}{(z_{\alpha}+ z_{\beta})}^{2} (1-\rho^{2})}{\left( \mu1-\mu2 \right)^{2}}$

Whereas $\sigma^{2} =\frac{\left( n1-1 \right){S1}^{2} +(n2-1) {S2}^{2}}{n1+n2-2}$

n = sample size;

Z _α_ = standard normal deviate for α, usually set with α = 0.05, α/2 = 0.025,

Z _0.05_ = 1.645, Z _0.025_= 1.96;

Z _β_ = standard normal deviate for β, which usually is set to 80% or 90%, β=0.2, β=0.1 respectively, Z _0.2_= 0.842, Z _0.1_= 1.282;

S1 = standard deviation of the post-test score in experimental group (S1 = 1.93);

S2 = standard deviation of the post-test score of control group (S1 = 2.28);

$\rho$ = correlation coefficient between pre and post test score of each outcome measure (Occiput-Wall Distance $\rho$ =0.9);

µ_1_ = mean of outcome measure after experimental group (Occiput-Wall Distance µ_1_ = 6);

µ_2_ = mean of outcome measure after control group (Occiput-Wall Distance µ_2_ = 7.88);

µ_1_-µ_2_  = mean difference between two groups;

- $n/\mathrm{group}=\frac{2\sigma^{2}{(z_{\alpha}+ z_{\beta})}^{2} (1-\rho^{2})}{\left( \mu1-\mu2 \right)^{2}}$
- $n=\frac{8.8098\times38.25631555856\times0.19}{3.5344}$
- $n=18.11991875310763$
- $n\approx18$

The dropout rate was set at 20% because the intervention period was 8 week which was a relatively long time, the final sample size was calculated as follows:

$n_{\mathrm{adj}}=\frac{n}{{(1-R)}^{2}}$

$n:Sample size$

$R:$ Rate of dropout

- $n_{\mathrm{adj}}=\frac{n}{{(1-20\%)}^{2}}$
- $n=28.125$
- $n\approx28$

Therefore, the total sample size of this experiment was 56.

## 3. Study design

This study used the randomized controlled trial, because it had a lot of advantages in clinical research. Each community had an equal opportunity to participate in either the treatment or control group. This minimized subjective selection and obfuscation bias in research.

This study used the randomized controlled trial design, which used two parallel groups, and used two elderly communities in Khon Kaen Province, Thailand. First, the elderly people were assessed for eligibility. If they were eligible, they were given the baseline evaluation. Two groups were created, 28 subjects in the ASE group (one community) and 28 in the control group (the other community). If the subjects completed the protocol, they were included in the analysis. Figure 3-1 is a flow chart representation of the study design.

Participants in the ASE group performed an 8-week ASE training. The training frequency was 3 d•wk^−1^, each training session should be guaranteed for at least 40 min•d^−1^ (ACSM’s, 2014). Every practice was led by coaches. During the ASE training: the 28 participants were divided into two groups; two coaches led them in the exercise. Participants in the control group stayed in normal life and did not participate in ASE training. The researcher gave the control group the same ASE training program at the end of the experiment.

## 4. Randomization

This research used random sampling to do the Cluster-Randomized Control Trial (RCT). A cluster randomized controlled trial is a type of randomized controlled trial in which groups of subjects (as opposed to individual subjects) are randomized. Cluster randomized controlled trials are also known as cluster randomized trials, group-randomized trials, and place-randomized trials. The Cluster-Randomized Control Trial was used, in this study, to avoid contamination between two groups of subjects. Random sampling reduced selection bias. It also increased the likelihood that each group in the study would accommodate the same number of participants. The two communities were randomly assigned to be the ASE group and a control group. The researcher used random sampling to select two communities with similar socio-economic status in Khon Kaen province, Thailand. Two envelopes were prepared by the researcher. One envelope contained ASE Group; and the other contained Control Group. The researcher randomly selected one envelope for each community. Once the groups were established, the participants were evaluated with Occiput-Wall Distance and other parameters as baseline values. With this Cluster-randomized controlled trial, levels of these baseline parameters between the two groups could not be guaranteed.

## 5. Experiment procedures

### 5.1 Ethical approval

Before participating in the study, all subjects were informed of the protocol and possible risks involved, as well as some issues to be noted in ASE intervention. They were informed orally and in writing before signing up to participate in the experiment. The proposal for the study took place after approval from the Ethical Committee of Khon Kaen University, Thailand (HE 612355).

### 5.2 Procedures

The researcher had a Thai research assistant. The Thai research assistant explained all the details for the participants on what they were expected to do during participation and answered all the questions that were asked by the participants. All the information was the same in all the documents.

The experiment was divided into two groups, the ASE group, the other was the control group. The whole experimental period was 12 weeks. The first two weeks were preparation: selecting the experimental subjects according to the inclusion and exclusion criteria, signing experimental protocols with participants, teaching participants the Arm Swing Exercise, and teaching them the whole training process. The formal experiment period was 8 weeks of intervention training.

Both groups wore loose, comfortable sportswear and flat shoes for exercise and data collection. The ASE group trained for 8-weeks, 3-time sessions per week, 40 min per-time session. The ASE training session had 3 parts: 5 min warm-up, 30 min main part training (ASE training), and 5 min cool-down. The training frequency was 3 d•wk^−1^, the training intensity was low-moderate intensity (Swain, 2014). Each training session was guaranteed for at least 40 min•d^−1^ (Swain, 2014). Participants in the control group stayed in normal life and did not participate in ASE training. The researcher will gave the control group the same ASE training program at the end of the experiment.

**Baseline Evaluation (n=92)**

**Subject not included (n=36)**

**Reason for ineligibility:**

-Age did not meet the experimental requirements (n=10)

-Cervical cancer (n=2)

-Physical disabilities (n=5)

-Family reasons (n=10)

-Unknown (n=9)

Randomized (n=56)

**ASEG (n=28)**

**CG (n=28)**

Immediate- complete experiment for day 1 (n=28)

Lost to follow-up: None

Week 4- complete experiment for 4 weeks (n=28)

Lost to follow-up: None

Week 8- complete experiment for 8 weeks (n=28)

Lost to follow-up: None

Subjects included in analysis (n=28)

Immediate- complete experiment for day 1 (n=28)

Lost to follow-up: None

Week 4- complete experiment for 4 weeks (n=28)

Lost to follow-up: None

Week 8- complete experiment for 8 weeks (n=28)

Lost to follow-up: None

Subjects included in analysis (n=28)

**Figure 3-1** Flow chart representation of the study design

SH

BADL

RSE

HR

BP

HRV

CSR

OWD

BS

TUG

STP

OWD

BS

STP

OWD

BS

STP

SH

BADL

RSE

HR

BP

HRV

CSR

OWD

BS

TUG

STP

**8-week experiment**

**1^st^ measure**

**Immediate (Day1)**

**2^nd^ measure**

**Baseline**

**Week 4**

**4^th^ measure**

**Week 8**

**3^rd^ measure**

**Figure 3-2** Outcome measurement processing: 1^st^ measure is baseline, 2^nd^ measure is immediate (after first time ASE), 3^rd^ measure is after four weeks, 4^th^ measure is after 8 weeks. Note: SH: Standing Height; BADL: Barthel Activities of Daily Living Index ; RSE: Rosenberg Self-Esteem scale; HR: Heart Rate; BP: Blood Pressure; HRV: Heart Rate Variability; CSR: Chair Sit-and-Reach; OWD: Occiput-Wall Distance; BS: Back Scratch; STP: Spatio-Temporal Parameter; TUG: Time Up and Go.

65

**Table 3-1**  The process of measurement (1^st^ and 4^th^ measure)

| **Measurement**  **sequence** | **Measurement time** | **Measurement frequency** | **Physical state during the test** |
| --- | --- | --- | --- |
|  | **Total time (30 min)** | **(time)** |  |
| 1. Occiput-Wall Distance | 2 min | 3 | Stand up |
| 2. Standing Height | 1 min | 1 |  |
| 3. Back Scratch | 2 min | 3 |  |
| 4. Heart Rate | 1 min | 1 | Sit down |
| 5. Blood Pressure | 1 min | 1 |  |
| 6. Heart Rate Variability | 5 min | 1 |  |
| 7. Chair-Sit-and-Reach | 2 min | 3 |  |
| 8. Spatio-Temporal Parameter | 4 min | 2 | Movement |
| 9. Time Up and Go | 3 min | 2 |  |
| 10. Barthel Activities of Daily Living Index scale | 5 min | 1 | Sit down |
| 11. Rosenberg Self-Esteem scale | 4 min | 1 |  |

**Table 3-2** The process of measurement (2^nd^ and 3^rd^ measure)

| **Measurement**  **sequence** | **Measurement time**  **(time)** | **Measurement frequency** | **Physical state during the test** |
| --- | --- | --- | --- |
|  | **Total time (8min)** | **(time)** |  |
| 1. Occiput-Wall Distance | 2 min | 3 | Stand up |
| 2. Back Scratch | 2 min | 3 |  |
| 3. Spatio-Temporal Parameter | 4 min | 2 | Movement |

Figure 3-2 is the outcome measurement process; it describes the process flow design of the whole experiment and the outcome to be measured. The experimental data plan measured four times, 1^st^ measurement was the baseline (before intervention training), 2^nd^ measurement was immediately (after doing the first time ASE), 3^rd^ measurement was after four weeks (before 5 fifth week), 4^th^ measurement was after eight weeks.

The first and fourth measurements were taken in the following sequence (Table 3-1 shows the process of 1^st^ and 4^th^ measurement): (1). Standing height (2). Barthel Activities of Daily Living Index. (3). Rosenberg Self-Esteem scale. (4). Heart Rate. (5). Blood Pressure. (6). Heart Rate Variability. (7). Chair Sit-and-Reach. (8). Occiput-Wall Distance. (9). Back Scratch. (10). Spatio-Temporal Parameter. (11). Time Up and Go. The second and third measurements were taken in the following the sequence (Table 3-2 shows the process of 2^nd^ and 3^rd^ measurement): (1). Occiput-Wall Distance. (2). Back Scratch. (3). Spatio-Temporal Parameter.

### 5.3 Management of experiment

Regulators in the elderly community control everything in the community, therefore this study needed to be approved by community managers before it was carried out. The researcher selected one leader for each of the 2 community groups. The leaders had knowledge of all of their own participants and were the managers of the volunteers and participants. The researcher used key people in the community to help us find reliable people for our experiment. All volunteers and leaders were trained before the experiment. Twelve volunteers were recruited before the experiment to manage all participants. The volunteers needed to be in good health.

Leaders organized the ASE training and managed the control group in an orderly manner. The leader and volunteers of the ASE group organized every training and measurement of outcomes. They also needed to be diligent that the exercise was done correctly and safely. Participants in the control group continued their normal life and did not participate in ASE training. The researcher conducted a basic situation survey once a week and gave the same ASE training program at end of the experiment.

The researchers in this study were coaches who had obtained a professional training certificate to ensure the accuracy of teaching the exercise. The coach taught the ASE to the leaders and volunteers before the experiment to make sure they had the right procedure. During the eight-week experiment, coach, leader, and volunteers led the older adults in practicing ASE together.

The ASE intervention called for at least 3 days of ASE, but we offered it 5 days a week so that people could get their 3 days a week as some participants could not attend one or two days during the week. Most participants considered these meetings as social events. On random days, small gifts were distributed. The participants received a small amount of money for their travelling expenses to participate.

### 5.4 Monitoring of ASE training

The researchers monitored the intensity of ASE training, by using a Polar stopwatch to measure exercise intensity. The American Heart Association generally recommends that the target heart rate be: vigorous exercise intensity: 70%-85% of maximum heart rate; moderate exercise intensity: 50%-70% of maximum heart rate; low exercise intensity: 40%-50% of maximum heart rate (Nelson, Rejeski, Blair, Duncan, Judge, King, et al., 2007). The ASE was 50-60% low-moderate exercise intensity of maximum heart rate in this study. The researchers measured heart rate on the ASE training; heart rate was 74±8 before ASE training, heart rate was 95±12 during ASE training (at 20-minute mark), heart rate was 91±8 after ASE training (40-minute marker). By monitoring participants’ heart rates, during the whole process of ASE, the average heart rate for older women was 87±9 beat per minutes. The ASE was reasonably and scientifically designed to control the exercise intensity of the elderly people. Metronomes and music are also used to control the rhythm of ASE for participants. This suggested that ASE was a safe and usefully exercise for older women.

The coach, leader and volunteers paid close attention to the physical condition of the participants during the ASE training. If it appeared that the elderly person was becoming physically ill, the participant would stop training immediately, and we would transport the participant to the hospital and have the doctor check the participant’s health status at that time.

## 6. Exercise protocols

**6.1 Warm-up program (5min)**

A dynamic warm-up is a quick and gentle way to prepare the body for ASE exercise. During the 5-minute warm-up period, the participant used almost every muscle and joint, from the neck to the ankles in the body.

Warm-up process included:

1. Neck Up and Down (30 seconds). Look up as high as possible and look down as far as possible, from the ceiling to the shoes.
2. Shoulder Circles (30 seconds). Move the shoulders up, then backwards, then down, and then forward circular fashion, trying to get the maximum range of shoulder motion.
3. Arm Swings Across (30 seconds). Arms should be straight, at a chest level horizontal line when swinging. Start with arms to the side and then cross in front of the body.
4. Arm Swings Forward (30 seconds). Arms are straight next to the body, swing forward, up as high as possible, then swing back behind the hips.
5. Wrist Circles (30 seconds). Put bent arms to 90 degrees, put elbows at sides, and make circles in front of body. Try to keep arms still and only move the wrist joints. Then reverse directions.
6. Trunk Twists (30 seconds). Arms are bent to 90 degrees and then rotating the spine, trying to look behind, then twist to the other side. Heels may come off the ground to allow it to become a whole-body twist.
7. Knee Bends (30 seconds). With feet firmly on the ground, the knees bend then moves forward around in circles. Then reverse directions.
8. Ankle Circles (30 seconds). Forefoot step on the ground, the ankle joint moves in a circle from front to back then reverse. Do each ankle separately.

**6.2 Arm Swing Exercise training program (30min)**

This study used the Chinese version of ASE. It is considered suitable for people aged 60-80 years (Kejin, 2014). The ASE training period was 30 min (Swain, 2014). All participants performed ASE in a hospital-provided activity area (the area is 200 square meters). The ASE steps include: The participants stood with their feet shoulder width apart. The arms were actively raised to shoulder-height with comfortably straightened fingers. While their trunk and neck were kept upright, their arms swung back and forth naturally following the preset tempo of the metronome. Breathing through their nose, they breathed in during the upswing and breathed out on the downswing. Each set of ASE consisted of five arm-swings, where the action from 1 to 4 was the same (Figure 3-3). However, on the fifth swing, the participants slightly bent their knees and dipped down twice ((Figure 3-3). Then they were back to the starting position. The participants were encouraged to swing their arms rhythmically in a relaxed manner (MeiMen YiQi Popular, 2018).

The researcher gradually adjusted the intensity of exercise. We used a metronome device and music to control arm swing speed. During the first 2 weeks of ASE training, the participants did a minimum of 15 Arm Swings per minute. During the 3-4 weeks of ASE training, the participants did a minimum of 20 Arm Swings per minute. During the 5-8 weeks of ASE training, the participants did a minimum of 20-25 Arm Swings per minute. There was be a two-minute break half way through during the 30 minutes of ASE (ASE process see Figure 3-3).


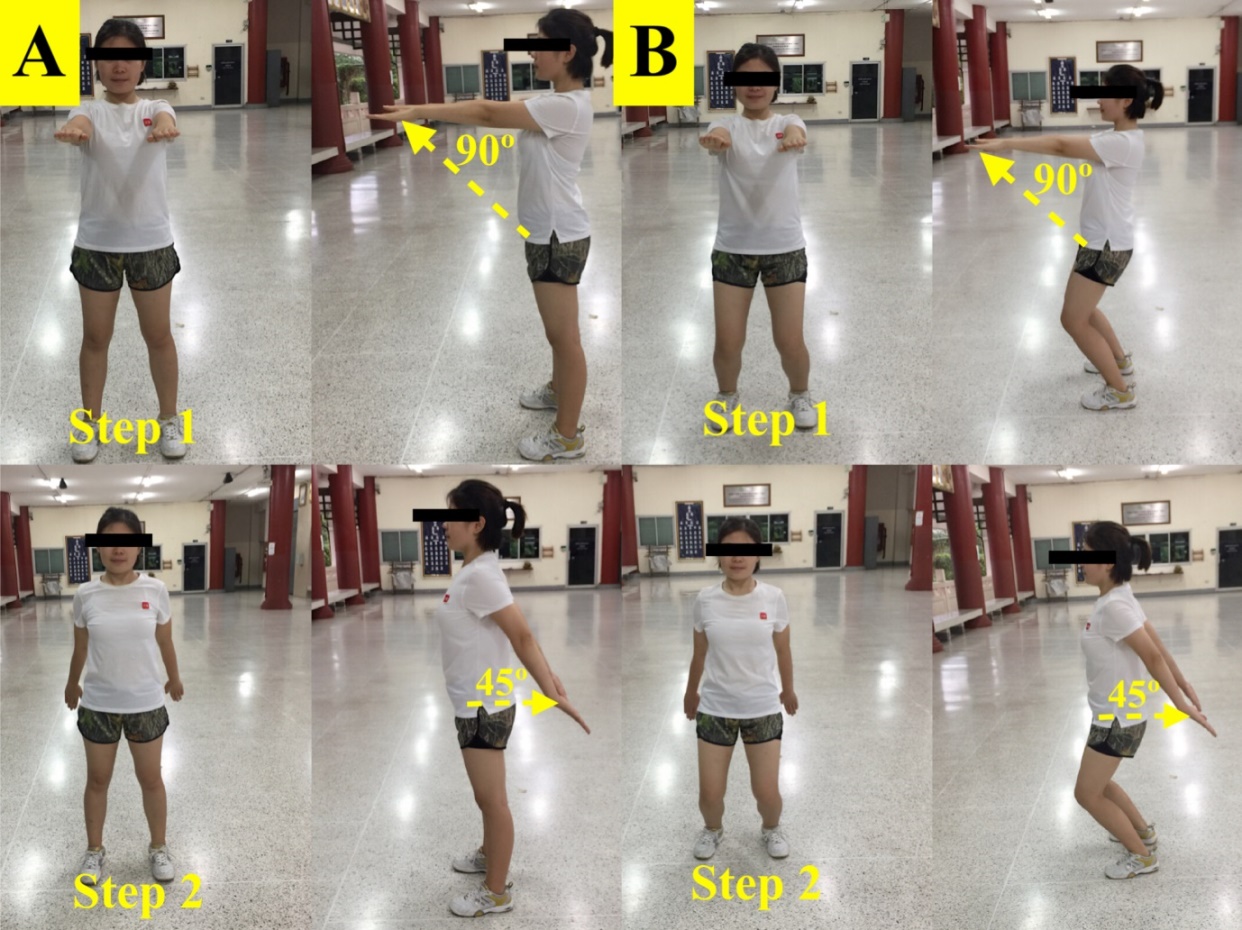


**Figure 3-3** Single set of Arm Swing Exercise performance consisted of the first four swings with knees extended (A) in the standing position and
the fifth swing (B) with slightly bent knees. The pictures were all taken by Dr. Xiao Zhen and who also prepared the Figure which has not been copyrighted.

**6.3 Cool-down program (5min)**

Cool-down was done after the exercise, letting the body gradually transition to a resting or near-resting state. Cooling down allowed the heart rate to return to its resting rate. It is effective in avoiding injury if performed after exercise.

1. Neck side stretch (50 seconds). Gently turn head to one side, then to the other side. Next lift the right arm up and over the head, resting palm gently on the left side. Gently pull head to the right. Hold for 20 seconds, then repeat on the other side.
2. Shoulder and upper back stretch (50 seconds). Begin by standing tall, arms by sides, reaching behind with both hands, pull shoulders back and clasp fingers together. Return to standing tall, and repeat.
3. Triceps stretch (50 seconds). Start by standing tall, lift right arm up over head, bending at elbow. Reach with the left arm up to clasp the other elbow and pull gently to the left. Hold for 20 seconds, then switch to do the other arms.
4. Back stretch (50 seconds). Start by standing tall, with hands on hips. Gently arc backward, looking up toward the ceiling. Hold for about three seconds, then return to standing. Repeat 10 times.
5. Ankle circles (50 seconds). Start by standing tall, begin to rotate right ankle; 10 to 20 rotations clockwise and 10 to 20 counter-clockwise. Repeat on opposite leg.

## 7. Outcomes measurements

A review of previous studies relating to the effectiveness of ASE on the elderly with posture and gait provided important information about both subjective and objective clinical outcomes. The results of this study were measured in four aspects: physiological, psychological, posture and gait. Before and after the training the following were measured: (1) Physiological Markers: Heart Rate (HR), Blood Pressure (BP) and Heart Rate Variability (HRV). (2) Psychological Marker: Rosenberg Self-Esteem Scale (RSE), Barthel Activities of Daily Living (BADL) Index (3) Posture Markers: Occiput-Wall Distance (OWD), Standing Height (SH), Back Scratch (BS) and Chair Sit-and-Reach (SR) (4) Gait Markers: Spatio-Temporal Parameter (STP) and Timed up and go (TUG).

**Reasons for choosing outcomes**

1. **Effective**

These outcomes truthfully reflected the purpose of the research design. They enabled researchers to obtain accurate and scientific results. The selected indicators verified the reliability and validity and can be used effectively in this study.

1. **Comprehensive**

Comprehensive assessment methods reflected the ASE intervention training effects of posture and gait on the elderly during a certain exercise more adequately than a single marker method (Finsterer et al., 2016).

1. **Easy to obtain**

Markers needed to be collected easily and cheaply. Measurements were easy to perform with widely available equipment (Finsterer et al., 2016).

### 7.1 Physiological markers

#### 7.1.1 Heart Rate (HR)

HR was measured to indicate the exercise intensity of the participants while doing the ASE exercise. Monitoring HR is one of the most commonly used means to evaluate the load in athletes. The use of HR monitoring during movement is based on the linear relationship between HR and the oxygen consumption during steady-state movement (Achten & Jeukendrup, 2003). Measuring the basic heart rate helped the researcher understand the state of the elderly and evaluate the effect of exercise. If the basic heart rate remained constant or gradually decreases, it indicated that the proper exercise load and body function were good. If the basic heart rate was unstable or high, it indicated that the exercise load was too high, and the fatigue of the body had not fully recovered. Therefore, the basic heart rate was a good parameter to evaluate exercise fatigue. The examiner used the Jumper JPD-500 (Figure 3-4), a pulse oximeter, to measure the resting heart rate of participants.

**Figure 3-4** Tool to test the HR (Jumper JPD-500). Image is not available.

#### 7.1.2 Blood Pressure (BP)

In this study, diastolic and systolic blood pressure measurements, before and after the experiment, were issued to verify the effect of ASE exercise on lowering blood pressure. Hypertension is a common cardiovascular disease; the elderly are more likely to suffer from hypertension. The prevalence rate is 40% ~ 60%, which is one of the major diseases seriously threatening the health of the elderly (Chobanian, Bakris, Black, & Cushman, 2003). In early times, high blood pressure was considered a physiological phenomenon that increased with age and did not require treatment. However, long-term studies have shown that elderly hypertension is an important factor that endangers the survival and quality of life of the elderly (Chobanian et al., 2003).

With each heartbeat, blood pressure varies between systolic and diastolic blood pressure. Systolic pressure, the peak pressure in the arteries, occurs near the end of the heart cycle when the ventricles contract (Booth, 1977). Diastolic pressure, which is the lowest pressure in the arteries, occurs when the heart cycle begins, and the ventricles fill up with blood. A resting healthy adult has a systolic blood pressure of 120 mm hg and a diastolic pressure of 80 mm hg which is an example of a normal measurement (Booth, 1977).

The automatic blood pressure monitor method was used to measure the blood pressure of the participants. It used an automatic blood pressure monitor (Figure 3-5) ; sleeves are comfortable and easy to use. Blood pressure measurement procedure:

1. Insert the arm into the band and bind it, measuring on bare arms; the pressure of the bundled arm band is moderate (Deakin, 2000).
2. The center of the brachial band is at the same height as the heart. The lower part of the arm band is 1-2cm from the elbow joint (Ostchega, Hughes, Zhang, Nwankwo, & Chiappa, 2013).
3. Relax palms while measuring, palms up, stay calm and relaxed during the measurement (Forouzanfar, Dajani, Groza, Bolic, & Rajan, 2011).

**Figure 3-5** Tool to test Blood Pressure. Image is not available.

#### 7.1.3 Heart rate variability (HRV)

The role of HRV in heart health during disease has long been studied. However, studies in recent years have shown that the HRV parameter changes at all stages of motion. These results show that HRV parameters can be used to analyze the pressure experienced by the body during and after training of the physiological recovery (Abramson, Rosenberg, Jewell, & Wright, 2007). In fact, autonomic nervous system changes reflected by HRV changes can be useful parameters for managing physical fatigue and controlling exercise intensity (Malik, Marek, 1998). HRV monitoring can help the elderly to develop personalized exercise training programs with reasonable monitoring of training load and recovery time (Acharya et al., 2007).

During ASE, the LF (low-frequency power) component increased, HF (high frequency power) decreased. So, the expected result after ASE intervention training: RMSSD (Root Mean Square of the Successive Differences) and HF increased. This means parasympathetic activity increased which reflected more relaxation.

Heart rate variability (HRV), the change of the continuous heartbeat over time, is predominantly dependent on the extrinsic regulation of the HR (Appel, Berger, Saul, Smith, & Cohen, 1989). HRV is a reliable reflection of many physiological factors that regulate the normal heart rhythm (Acharya et al., 2006). During testing, walking velocity at heart rate variability threshold was registered. The correlation between walking velocity -HRV 1 and walking velocity -HRV 2 was significant (r = 0.84); the infraclass correlation coefficient was high (0.92; 0.82 to 0.96) (Dourado & Guerra, 2013).

Data collected for this experiment was: HRV data baseline and after the experiment, power spectrum analysis recorded in static sitting position as well as recording HRV data from the finger for 5 minutes. The equipment was connected to the test software (UBiomacpa) (See Figure 3-6) of the computer terminal by connecting the test device on a middle finger. The test environment was in a quiet lab, without intense light and noise, participants had avoided caffeine two hours before the experiment, and avoided strenuous exercise two hours before the experiment. Subjects were measured in a comfortable sitting position during the experiment. Participants remained quiet and awake during the test. During the whole test, the subjects’ breathing was directed to be natural breathing.


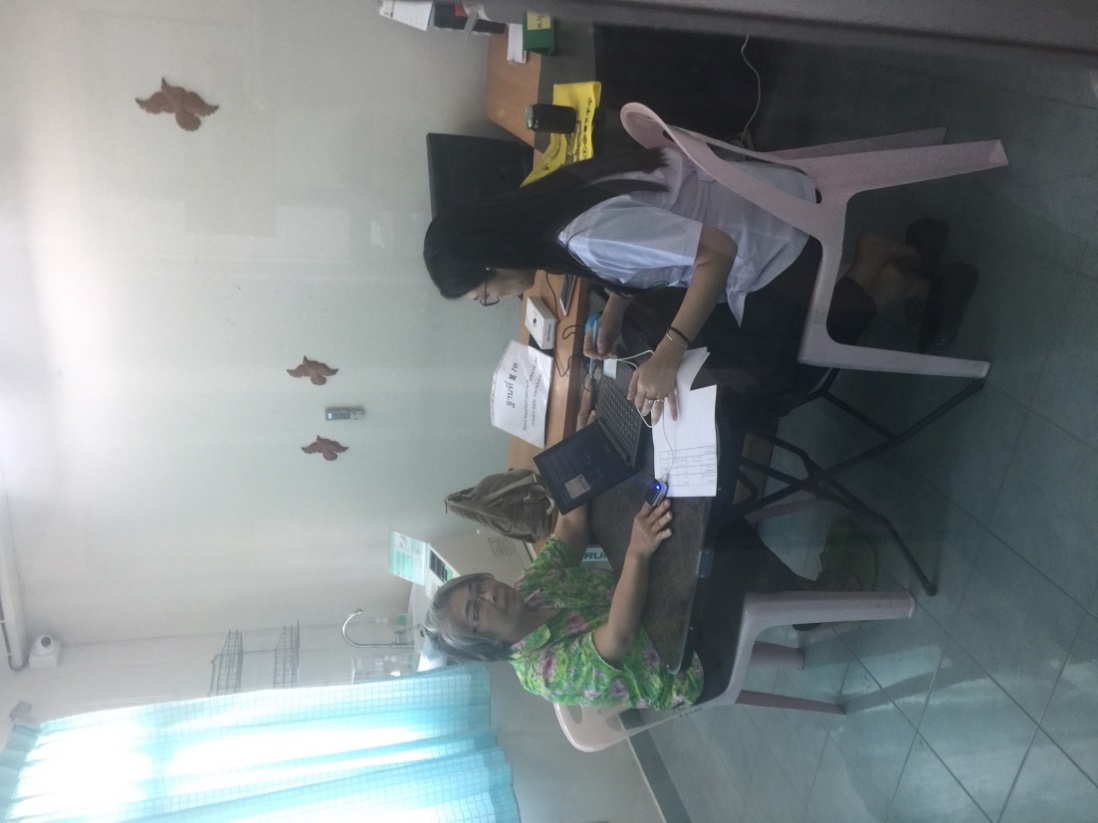


**Figure 3-6** Measurement of HRV. The picture was taken by Dr. Xiao Zhen and has not been copyrighted.

### 7.2 Psychological marker

#### 7.2.1 Rosenberg Self-Esteem Scale (RSE)

The RSE scale was used to evaluate the self-esteem of the elderly. This study explored whether there will be any change in the self-esteem scores of the elderly before and after the intervention training, to determine whether ASE exercise enhanced self-esteem in the elderly (Danner, Snowdon, & Friesen, 2001). As people gradually get older, their mental state will gradually change with symptoms like depression or anxiety. These factors also indirectly affect the elderly’s posture and gait, so it could have an impact on their physical health, cognition, interpersonal relationships and even survival (Danner et al., 2001).

The Rosenberg Self-Esteem Scale (RSE) was developed by sociologist Dr. Maurice Rosenberg as a widely used measure of self-esteem in social science research. It uses a 0-30 scale, and a score below 15 may indicate a lack of self-esteem (Blascovich, & Tomaka, 1991). The RSE scale’s design is similar to that of social questionnaires, it includes four answers to every question: 0= Strongly disagree, 1= Disagree, 2= Agree, 3=Strongly agree. The respondents, through this scale, were asked how they currently feel to measure their self-esteem (Blascovich, & Tomaka, 1991).

The Rosenberg Self-Esteem Scale (RSE) was used to measure the elderly’s psychological state. The reliability of the scale was satisfactory (r = .88) with no gender or age difference (Chen, Fan, & Moe, 2002). The Cronbach’s Alpha in the Thai version of the Rosenberg Self-Esteem Scale was 0.849 and proved to have reliability and validity. It provided an effective measure of self-esteem. It can be used for effective and reliable testing of the mental state of the elderly (Piyavhatkul, Aroonpongpaisal, Patjanasoontorn, Rongbutsri, Maneeganondh, & Pimpanit, 2011).

The Thai version of the Rosenberg Self-Esteem Scale was used in this experiment. For items marked with an (R), reverse the scoring (for example, 0 (Strongly disagree) = 3, 1 (Disagree) = 2, 2 (Agree) = 1, 3 (Strongly agree) = 0). Add a score to the space next to the question. The typical score on the Rosenberg scale is about 22 points, most people scoring between 15 and 25 points. Low self-esteem can be a problem if the score is below 15 (callhelpline, 2010).

#### 7.2.2 Barthel Activities of Daily Living Index (BADL)

Normal aging can reduce the functional status of the elderly. The decline in age may trap the elderly in a vicious iatrogenic circle, leading to further health problems with activities of daily life. The deterioration of posture and gait in older adults is closely related to their ability to perform activities of daily living. Good posture and gait in the elderly help them have good activities of daily living. The intervention experiment was used to assess whether the activities of daily living of the elderly improved due to the intervention training.

The BADL is an ordered scale that is used to measure the daily life activities of the elderly. The index is an ordered scale consisting of ten daily activities which includes: mobility indoors, transfers, stairs, toilet use, bladder, bowels, bathing, grooming, dressing and feeding. It uses the ten variables to describe BADL and mobility. The higher score was associated with a greater likelihood of being able to live independently at home (Sherwood, Mor, V., & Morris, 1977). Evaluation of BADL may identify the early decline in older adults or other competent and healthy individuals. It is a simple and effective tool for evaluating the functional abilities of older adults (Hokoishi, Ikeda, Maki, Nomura, Torikawa, Fujimoto, et al., 2001). The BADL is a valuable comprehensive assessment scale (Hartigan, 2007).

The BADL has a high inter-rater reliability correlated between the patient’s self-reported consistency (0.99) (Sherwood, Mor, & Morris, 1977), and test-retest reliability (0.89) (Sainsbury, Seebass, Bansal, & Young, 2005) (see Figure 13). This study used the Thai version of the Barthel ADL Index Scale. It has a high correlation with the Barthel Index scores (r = 0.80) (Senanarong, Harnphadungkit, Prayoonwiwat, Poungvarin, Sivasariyanonds, Printarakul, et al., 2003). The Thai version (see Figure 14) of the BADL is easy to use and has high reliability.

### 7.3 Posture markers

#### 7.3.1 Occiput-Wall Distance (OWD)

OWD is an index of kyphosis (Graham, Middleton, Roberts, Mallinson, & Prvu-Bettger, 2018). The association between OWD and measurements of physical performance were evaluated (Antonelli-Incalzi, Pedone, Cesari, Di Iorio, Bandinelli, & Ferrucci, 2007). The flexed posture defined by OWD is also associated with increased posterior chest convexity. It has been widely used in epidemiological studies, OWD can be used as a simple tool to screen and monitor the severity of kyphosis (Nishiwaki et al., 2007). It could be a good screening tool for early detection of forward neck posture and possible weakness of posterior neck muscles in non-disabled elderly people (Szucs & Brown, 2018). Changes of posture may cause kyphosis which is associated with postural instability, osteoporosis, disability and depression. The relationship between OWD and physical performance measurements was assessed (Antonelli-Incalzi et al., 2007). In males, longer OWD led to decreased balance and walking speed; in females, OWD is associated with decreased walking speed (Antonelli-Incalzi et al., 2007).

The results of one study indicated that there is a strong relationship between OWD and Flexicurve (r = 0.902, p<0.001), thus confirming the concurrent validity of OWD (Antonelli-Incalzi et al., 2007). This index has high reliability and validity.

As the participants stood up erect where the heels, sacrum, and upper back were positioned against the wall (Antonelli-Incalzi et al., 2007)， the examiner used a rigid ruler to measure the distance between the protuberance of the 7th cervical spinous process (see Figure 3-7, 3-8) (Siminoski, Warshawski, Jen, & Lee, 2011; Wiyanad, Thaweewannakij, Wattanapan, & Sooknuan, 2017).


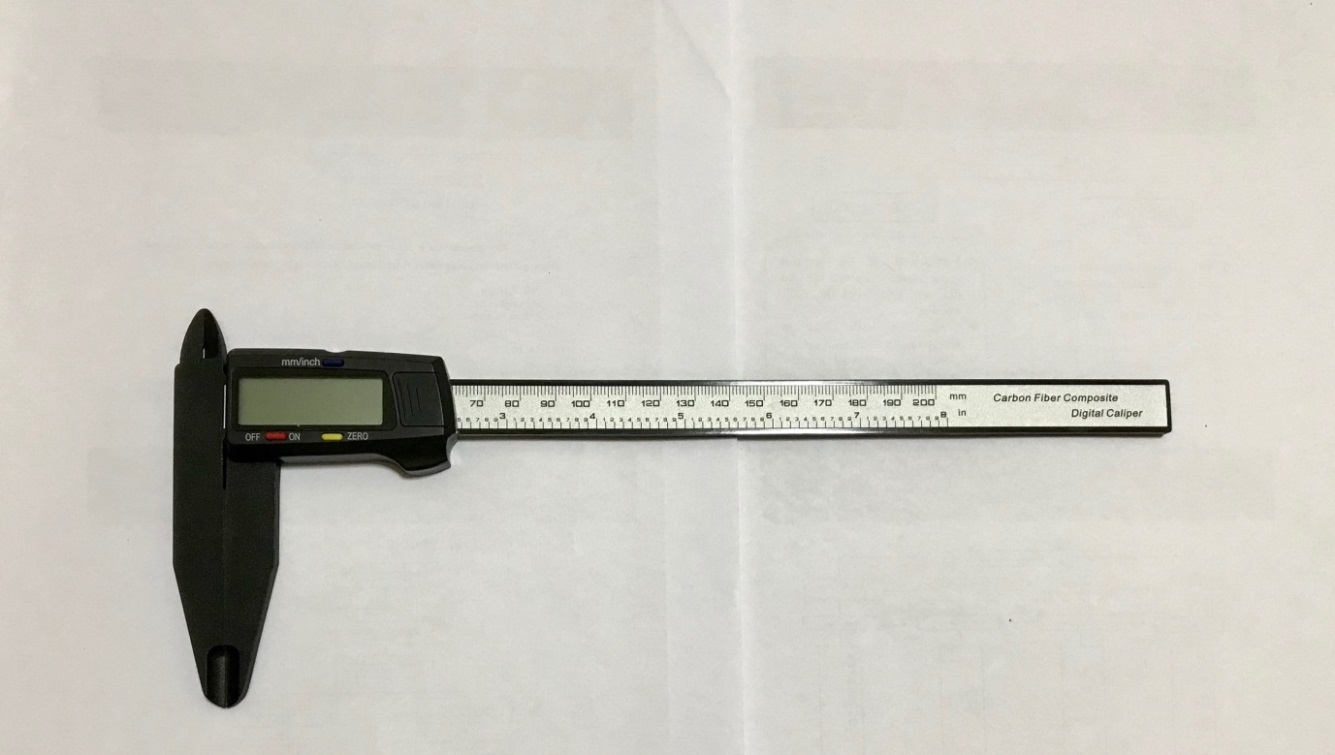


**Figure 3-7** Tool test of Occiput-Wall Distance. The picture was taken by Dr. Xiao Zhen and has not been copyrighted.


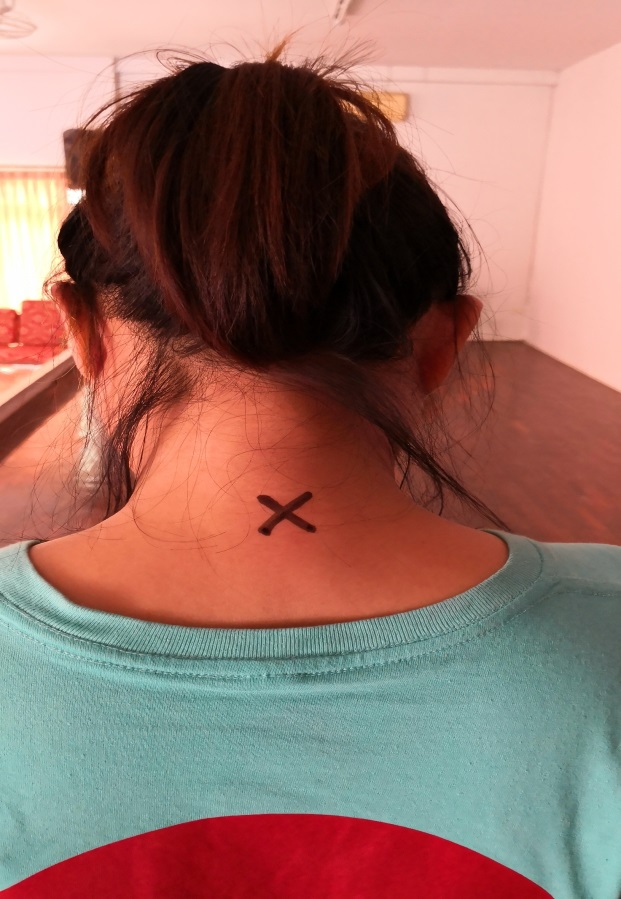

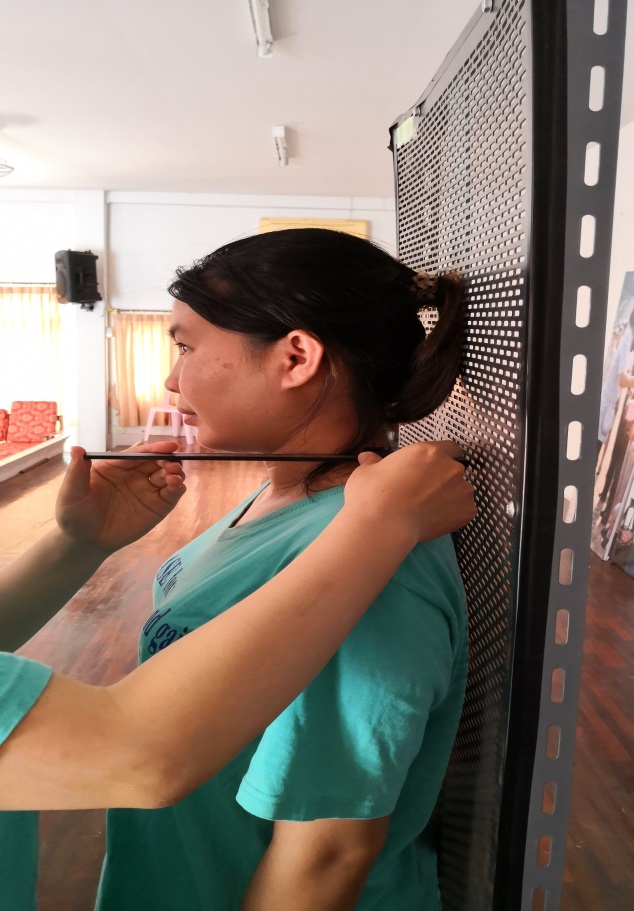


**Figure 3-****8** Measurement of Occiput-Wall Distance. The pictures were taken by Dr. Xiao Zhen who prepared the Figure and which has not been copyrighted.

#### 7.3.2 Back Scratch

The back scratch test measures the distance between the hands on the back, which measures the range of motion of the shoulders and the upper body and shoulders’ flexibility (Rózanska-Kirschke, Kocur, Wilk, & Dylewicz, 2006). It is closely related to the upper body posture of the elderly, where good upper body flexibility and large range of motion can maintain good posture. It is associated with older people’s lifestyles. This is important in tasks like grooming, dressing, reaching for things, putting on your overhead clothes and reaching for your seat belt (Dobek, White, & Gunter, 2006).

By testing the back-scratch test on patients with fibromyalgia in middle-aged and elderly women, the reliability and feasibility of the index were verified. Intra-class correlation coefficients (ICC) were 0.96 in the back-scratch test (Carbonell-Baeza et al., 2015).

Participants were tested for “back scratches” while in a standing position. By putting one hand over the shoulder on the same side and the fingers were extended as far down as possible. With the other hand on the back, with the palm facing the outside, fingers extended upward, trying to grab the fingers of the other hand (Rózanska-Kirschke et al., 2006). Align the fingers and measure the distance between the tips of the middle fingers. The tester instructed the participant how to adjust the position of the hands so that the middle finger may be close (Dobek et al., 2006). Care must be used to not scratch or pull participants’ fingers. The test used a 30-centimeter ruler. Both sides were tested twice. The difference between the two middle fingers was the result of the experiment, if the fingers overlapped, the value was positive – “+”, otherwise negative – “-”. The best score is recorded to the nearest centimeter (Rózanska-Kirschke et al., 2006). The average of the two measurements in a row, the measurement was accurate to 0.5cm.

#### 7.3.3 Chair Sit and Reach Test (CSR)

Chair Sit and Reach is a lower body flexibility trial. It can be used to evaluate effectively the flexibility of the elderly’s lower body. Good flexibility is an important factor in posture. A study examined the Chair Sit-and-Reach Test’s retest reliability and validity as a measure of hamstring flexibility in the elderly. The results showed that CSR has good intraclass test-retest reliability (r = .92 for man; r = .96 for woman) (Jones, Rikli, Max, Noffal, Jones, Rikli et al., 1996). This suggested that CSR is a safe test that can be a highly effective measurement of hamstring flexibility in the elderly.

Participants sat at the front of a chair, one leg extended forward to the ground (knee straight, heel on the floor and the ankle bent at 90°) and the other leg bent at the knee, hands reaching toward toes (Dobek et al., 2006) (see Figure 3-10). The trial involved anterior flexion to keep the spine as straight as possible with the head located on the spine axis. Arms were extended forward, and hands were together. The participants were instructed to inhale, and as they exhale (Dobek et al., 2006) they were to reach forward towards their toes and bend hips, keeping their back straight and head up. They Kept knees straight and stretched for 2 seconds. They avoided fast and vigorous exercise during the test, and the pain threshold should not be exceeded (Jones et al., 1996). The distance was measured between the tip of the middle finger and the first toe. If the fingertips touched the toes, the score was zero. The positive values “+” indicated the fingers crossed the toes line, the negative “-” value indicated the distance between the fingers and toes. The average of two measurements was taken in a row, and the measurement was accurate to 0.5cm (Rózanska-Kirschke et al., 2006).

#### 7.3.4 Standing Height (SH)

This study through the ASE intervention exercise was to verify whether it can enhance the elderly’s standing height. A person’s height is the distance from the bottom of his feet to the top of the head, measured with a stadiometer, using the metric system (Lapham, & Agar, 2012). When people get older, the discs between the vertebrae dry out and become thinner, which causes the spine to become compressed. As they get older they might get cone compression fractures, a wedge change in the cone results in a reduction in height and kyphosis. Osteoporosis can also cause bone loss (Samaras, 2012).

Measurements were taken at the same time (more accurate in the morning), using the same height measuring equipment with consistent body posture. Height measuring equipment was laid flat on the ground and against the wall. Two measurements was taken in a row. After the first measurement, measure again 30 seconds later; the results of both measurements should be approximately the same. The error of the two measurements should not exceed 0.5 cm (Voss, Baileey, Cumming et al., 1990). The subjects were barefoot, standing upright on the base of the altimeter, with the heel, sacrum, and between the shoulder blades resting on the column of the altimeter. The measurer stood on the left or right of the person being measured. The head was adjusted to the lowest point of the upper lobe of the tragus and the lower rim of the orbit. The level board of the altimeter was moved to the top of the head of the subjects being measured (see Figure 3-9).

**Figure 3-9** 2-meter wall mounted height measure measuring tape. Image is not available.

### 7.4 Gait markers

#### 7.4.1 Spatio-Temporal Parameter

This study used the “BTS G-WALK” gait analysis system (see Figure 3-10) to measure the Spatio-Temporal Parameter. G-WALK is a new approach to motion analysis: a specialized wireless inertial sensor applied to the patient enables the clinician to perform clinical tests. It also supplies the most relevant parameters related to walking, running, and jumping. G-walk is a portable device for motion analysis. This computerized gait cycle analysis can be performed on a variety of surfaces. It is accurate, fast and light, and is easy to execute (Paolo Gaffurini, 2013), and the results can be compared directly to the “normal” score based on the integration protocol. The BTS G-walk gait analysis system has been used for the following evaluation: speed, cadence, step length, stride length, gait cycle duration, stance phase duration, swing phase duration and double and single support duration (Paolo Gaffurini, 2013).

**Figure 3-10** Tool to test Spatio-Temporal Parameter. Image is not available.

Gait analysis based on the centroid system is an effective method to evaluate the effect of clinical intervention (Park, G., & Woo, Y. 2015). The researcher uses the G-WALK to measure the elderly’s speed, cadence, step length, stride length, gait cycle duration. The effect of ASE training on the gait of the elderly is determined by evaluating the gait Spatio-Temporal Parameters before ASE, immediately, 4week, and after 8week.

G-walk is a wearable device, it is attached to a special belt; the patient is free to walk, run and jump. The sensor sends all data to a computer connected via Bluetooth; at the end of each analysis, it displays an automatic report which contains all parameters recorded during the test (Park, G., & Woo, Y. 2015). Participants wear the device in the position of the sacrum S1. Participants maintain a normal walking speed on a 20-meter track. Measurements are taken 2 minutes at a time for a total of 4 minutes to get a measurement of gait Spatio-Temporal Parameters.

#### 7.4.2 Timed Up and Go (TUG)

The Timed Up and Go test has been used to identify risk in older adults due to balance or gait problems (Podsiadlo, & Richardson, 1991) and to measure progress in balancing, sitting and walking. TUG is a simple test to assess the mobility of older people, measure balancing and functionality. It was originally developed as a clinical measure of balance for the elderly, rated l to 5, based on the observer’s perception of the risk of falling during the test (Podsiadlo, & Richardson, 1991). The TUG is a reliable and effective test, which can be used to quantify functional mobility, and can also be used for clinical changes. This test is easy to learn, convenient for application, safe and reliable, no special equipment or training is required, and it can easily be included in routine medical examinations (Large, Gan, Basic, & Jennings, 2006).

In one study, the intraclass correlations were greater than 0.90, were similar within and between raters of TUG and had high intraclass correlation coefficients in elderly populations (Nordin, Ellinor, & Rosendahl, 2006), and its construction coefficient showed that TUG scores are related to gait speed (Pearson r = .75) (Peters, Fritz, & Krotish, 2013). TUG can be used in clinical evaluation and research. The TUG is a simple test to assess a person’s ability to move, which requires static and dynamic balance (Herr & Grabowski, 2012).

Equipment requirements: armchair - approximate seat height = 43-46 cm and approximate arm height = 65 cm, measuring tape which measures the distance of 3 meters, a line on the floor 3 meters from the chair (participants may use their daily walking aid tool) (Podsiadlo & Richardson, 1991), and stop watch. The participant, wearing comfortable shoes, sits in an armchair, leaning back, with arms on the arms of the chair. From the starting position, the participant stands, walks 3 meters (there are no obstacles when walking.), turns around, walks back to the chair, and sits down (Podsiadlo & Richardson, 1991).

The process included 5 steps: step 1: stand up, step 2: walk 3 meters, step 3: turn around, step 4: walk 3 meters, step 5: sit down. Assessor: start clock when “go” is said; stop the timer when the participant sits down; the criterion for sitting is that the hips touch the chair. The data was measured two times; participants were allowed one practice walk with the experimental results recorded during the 2nd trial. Instructions: on the word “go”, participants get up, walk to the line on the floor at a comfortable, safe pace, turn around, go back to the chair and sit down (Alberta Health Services, 2009).

##

## 8. Statistical analysis

The outcome was analyzed as a continuous variable and expressed as mean ± Standard Deviation (SD). The aim of the study was to understand the effect of ASE on the gait and posture of the elderly. The analysis of covariance (ANCOVA) was used for statistical analysis to compare the differences of the two groups at baseline, immediately, after 4 weeks and after 8 weeks. One-Way Repeated-Measures ANOVA was used for statistical analysis to compare the differences within groups at baseline, immediately, after 4 weeks and after 8 weeks. The differences between the ASE group and control group in the 95% confidence interval were calculated. The researcher used Statistical Package Social Sciences software for statistical analysis (version 26.0 IBM, Armonk, NY, USA； downloaded from Khon Kaen University), P<0.05 was considered statistically significant.
