# Supplementary material for: The benefits of Shuai Shou Gong (SSG) demonstrated in a Randomised Control Trial (RCT) study of older adults in two communities in Thailand
Source: PLoS One. 2023 May 25;18(5):e0282405. doi: 10.1371/journal.pone.0282405 (PMC10212083; doi:10.1371/journal.pone.0282405)
Supplement: S2 Data — (DOCX) [file pone.0282405.s008.docx]

**The Benefits of Shuai Shou Gong (SSG) Demonstrated in a Randomised Control Trial (RCT) Study of Older Adults in Two Communities in Thailand *[PONE-D-20-37977]***

The description of entries in file SSG_database.xlsx is as follows:

ID anonymised subject identifier
group 1 is Exercise Group and 2 is Control Group participant

C7WD C7 to Wall Distance

SH Standing Height

BSL Back Scratch of Left arm

BSR Back Scratch of Right arm

CSRL Chair Sit and Reach of Left leg

CSRR Chair Sit and Reach of Right leg

TUG Timed Up and Go

BADL Barthel Activities of Daily Living index

RSES Rosenberg Self Esteem Scale

For the outcome measures, 1 refers to baseline, 2 to the measure recorded after one session of SSG, 3 to the measure recorded after 4 weeks and 4 to the measure recorded after 8 weeks.
